# Role of Divalent Ions in Membrane Models of Polymyxin-Sensitive and Resistant Gram-Negative Bacteria

**DOI:** 10.1021/acs.jcim.4c01574

**Published:** 2025-01-18

**Authors:** Mariia Savenko, Robert Vácha, Christophe Ramseyer, Timothée Rivel

**Affiliations:** † Institute of Organic Chemistry and Biochemistry of the Czech Academy of Sciences, Prague16000, Czech Republic; ‡ Laboratoire Chrono-Environnement UMR CNRS 6249, Université de Bourgogne Franche-Comté, Besançon25000, France; § Central European Institute of Technology, Masaryk University, Brno60200, Czech Republic; ∥ National Centre for Biomolecular Research, Faculty of Science, Masaryk University, Brno60200, Czech Republic

## Abstract

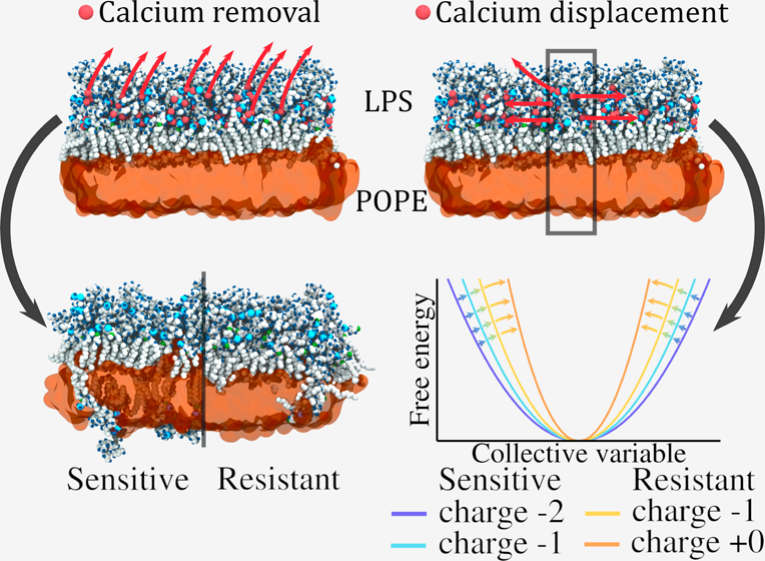

Polymyxins, critical last-resort antibiotics, impact the distribution of membrane-bound divalent cations in the outer membrane of Gram-negative bacteria. We employed atomistic molecular dynamics simulations to model the effect of displacing these ions. Two polymyxin-sensitive and two polymyxin-resistant models of the outer membrane of *Salmonella enterica* were investigated. First, we found that the removal of all calcium ions induces global stress on the model membranes, leading to substantial membrane restructuring. Next, we used enhanced sampling methods to explore the effects of localized stress by displacing membrane-bound ions. Our findings indicate that creating defects in the membrane-bound ion network facilitates polymyxin permeation. Additionally, our study of polymyxin-resistant mutations revealed that divalent ions in resistant model membranes are less likely to be displaced, potentially contributing to the increased resistance associated with these mutations. Lastly, we compared results from all-atom molecular dynamics simulations with coarse-grained simulations, demonstrating that the choice of force field significantly influences the behavior of membrane-bound ions under stress.

## Introduction

The growing occurrence of multidrug-resistant (MDR) Gram-negative bacteria poses a significant threat to public health.^1−3^ Of particular concern is the resistance to last-resort antibiotics, such as those in the polymyxin family, which are crucial for treating infections caused by these resilient pathogens. Despite extensive efforts from the scientific and political institutions to develop novel antibiotic therapies, the approval rate for new drugs has been alarmingly low in recent years.^4−6^ This trend is exacerbated by a limited understanding of the dynamic changes within the cell envelope of Gram-negative bacteria under environmental stresses.

The Gram-negative cell envelope, comprising an inner membrane (IM) and an outer membrane (OM) separated by a peptidoglycan layer, plays a fundamental role in protecting bacteria from external threats, including antibiotics. The OM, with its unique asymmetric structure featuring phospholipids on the inner leaflet and lipopolysaccharides (LPS) on the outer leaflet, significantly contributes to the low permeability of Gram-negative bacteria to drugs.^7−9^ The presence of integral proteins enriches the functions of this membrane^10,11^ and plays an important role in the modes of action of some antibiotics.

In this work, we focus on the interactions of antibiotics with the lipid matrix of the OM, particularly with LPS. LPS are amphiphilic molecules that expose a substantial hydrophilic layer, including O-antigen and parts of core oligosaccharides.^12−14^ This hydrophilic layer interacts with the external environment, which is crucial for bacterial survival under stress conditions such as antibiotic exposure.^15^ Modifications in LPS structure enable bacterial resilience to antibiotics, contributing to the diverse chemotypes and adaptive resistance mechanisms observed in Gram-negative bacteria.^16,17^

Among the limited arsenal of effective antibiotics against MDR Gram-negative bacteria, polymyxins B (PMB) and E (PME, colistin) stand as last-resort treatments.^18−21^ Initially relegated due to their nephro- and neurotoxic effects,^22,23^ polymyxins have regained prominence in combating antibiotic-resistant infections.^15,24^ Their unique structure, consisting of a cyclic heptapeptide linked to a fatty acid tail by a central tripeptide, facilitates specific interactions with the OM, particularly by means of their positively charged diaminobutyric acid (DAB) residue. The cyclic peptide structure is also critical for their antibacterial activity, distinguishing them from less effective linear analogs.^21,25,26^

The mechanism by which polymyxins exert their bactericidal effect involves multiple stages of interaction with the OM,^13,15,27−31^ some of which remain under debate. Upon contact, polymyxins bind to the OM surface, which is facilitated by their cationic nature. Polymyxins accumulate in regions of membrane defects,^27,32^ i.e., regions involving local modifications in membrane structure, although the exact nature of these membrane defects and their impact on polymyxin action when adsorbed to the membrane are still unclear. Previous works have indicated that polymyxin B may aggregate in solution^33^ and on the surface of OM models, forming micelle-like structures.^27,28,32^ However, it is unclear whether polymyxin aggregation is necessary for an effective bactericidal effect.

Following adsorption to the OM, polymyxins are believed to superficially insert their fatty acid chain into the membrane.^21^ This partial insertion is presumably made possible by the combined presence of the fatty acid chain,^25^ the cyclic peptide structure, and the presence of the cationic DAB residues. It is believed that these residues displace membrane-bound divalent cations,^15,18,20,21,23,34^ i.e., Ca^2+^ or Mg^2+^. This displacement weakens the OM structure, potentially allowing polymyxins to penetrate deeper into the lipid bilayer and exert their antibacterial effects.

However, bacterial resistance to polymyxins has emerged as a growing concern, necessitating a deeper understanding of the resistance mechanisms employed by Gram-negative bacteria. Resistance often involves alterations to LPS, such as the addition of chemical groups like phosphoethanolamine (PEtN),^31,35−37^ 4-aminoarabinose (Ara-4N),^31,35,36^ or galactosamine (GalN),^38^ which tends to neutralize the overall negative charge of LPS,^39^ diminishing repulsive interactions with polymyxins and reducing their reliance on the presence of divalent ions. Understanding the implications of these resistance mechanisms for the interactions between polymyxins and the OM is critical for developing strategies to combat polymyxin resistance.

To elucidate these intricate processes, molecular dynamics (MD) simulations provide a powerful tool for studying atomic-scale interactions and dynamics over submillisecond time scales. Recent studies have highlighted conflicting findings regarding the influence of polymyxins on membrane-bound divalent cations.^27,40−43^ While some authors see long-term direct ion displacement as a consequence of polymyxin binding, others report mainly induced membrane curvature, especially for OM models based on either lipid A or deep rough LPS. These variations are influenced by factors such as the choice of force field, the LPS model, and the concentration of polymyxins. These discrepancies highlight the need for further investigation to clarify the precise mechanisms of polymyxins’ action and development of resistance against it.

In this work, we employ a computational approach to investigate how ion displacement affects OM properties and facilitates polymyxin internalization. Through all-atom simulations, we model a global stress involving the removal of all divalent ions in both colistin-susceptible and colistin-resistant model membranes of *Salmonella enterica*. Additionally, we introduce a novel collective variable to model local stress on the divalent ions that could be induced by polymyxins. Finally, we applied this collective variable to coarse-grained simulations, which can reach longer time scales.

## Results

### Reaction of the OM to Removal of Divalent Ions

**Figure 1:**
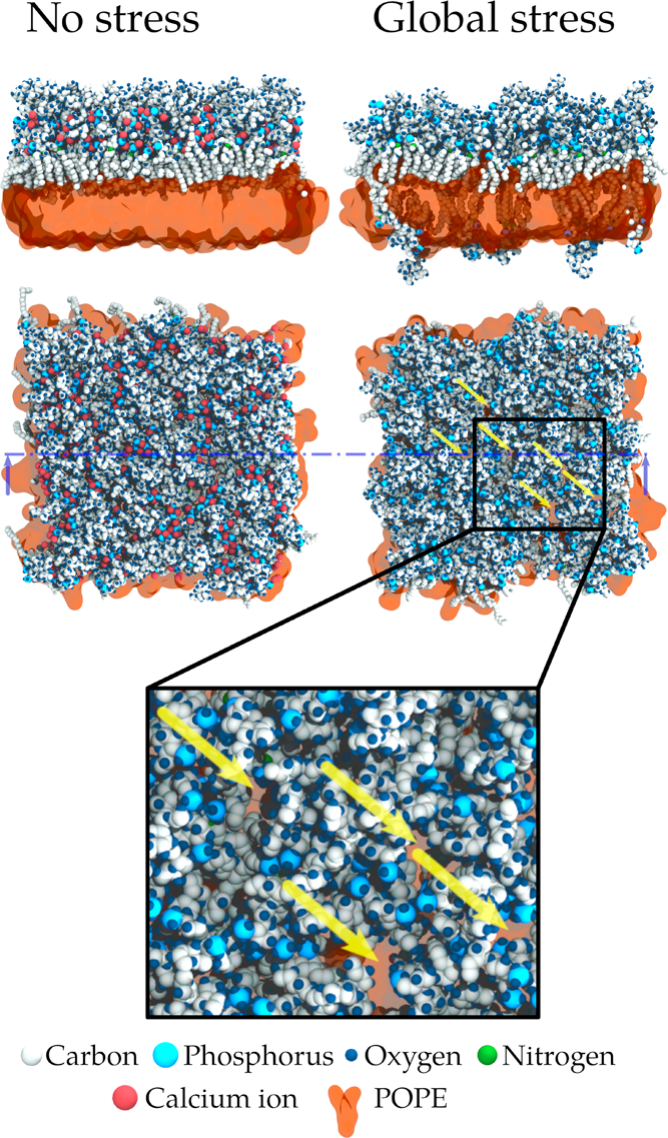
Representative snapshots from our simulations of one model of outer membrane of Gram-negative bacteria (system P2), in the absence of stress and under a global stress originating from the removal of all calcium ions in the simulation box (and their replacement by sodium in the solvent). Global stress induces the formation of cracks in each leaflet. Yellow arrows show some of these cracks that reside in the LPS leaflet.

Divalent ions, particularly Ca^2+^ and Mg^2+^, play a bridging role between LPS of the OM of Gram-negative bacteria. To better understand the impact of their displacement on membrane properties, we first modeled the global removal of these ions for two polymyxin-sensitive and two polymyxin-resistant models. Figure 1 shows that global stress creates major membrane remodeling, including LPS flipping as well as cracks—that are defined as a partial membrane discontinuity—^44^at the surface of the LPS layer in the polymyxin-sensitive model membrane referred to as P2.

The signature of calcium ion removal is clearly recognizable in Figure 1, in our simulations relying on the CHARMM36 force field.^45^ Indeed, on the side view of the system with no stress applied, a first layer of phosphorus atoms and calcium ions, associated with the lipid A phosphate groups, and a second larger layer, associated with LPS inner core phosphate groups, are recognizable (see the density profile in Figure S1 as well), while this structure is clearly hampered in the system where all divalent ions were removed and where the membrane profile looks rougher (see also Figures S2–S5). Following this observation, we analyzed membrane curvature and fluctuations. We used the SuAVE package^46^ to assess whether the membranes remain flat. We computed the distribution of the surface angle (Figure S6) for our model membranes in the absence of stress and under global stress. We observed that the maximum likelihood angle is between 4 and 7°, meaning that the membrane remained flat.

**Figure 2:**
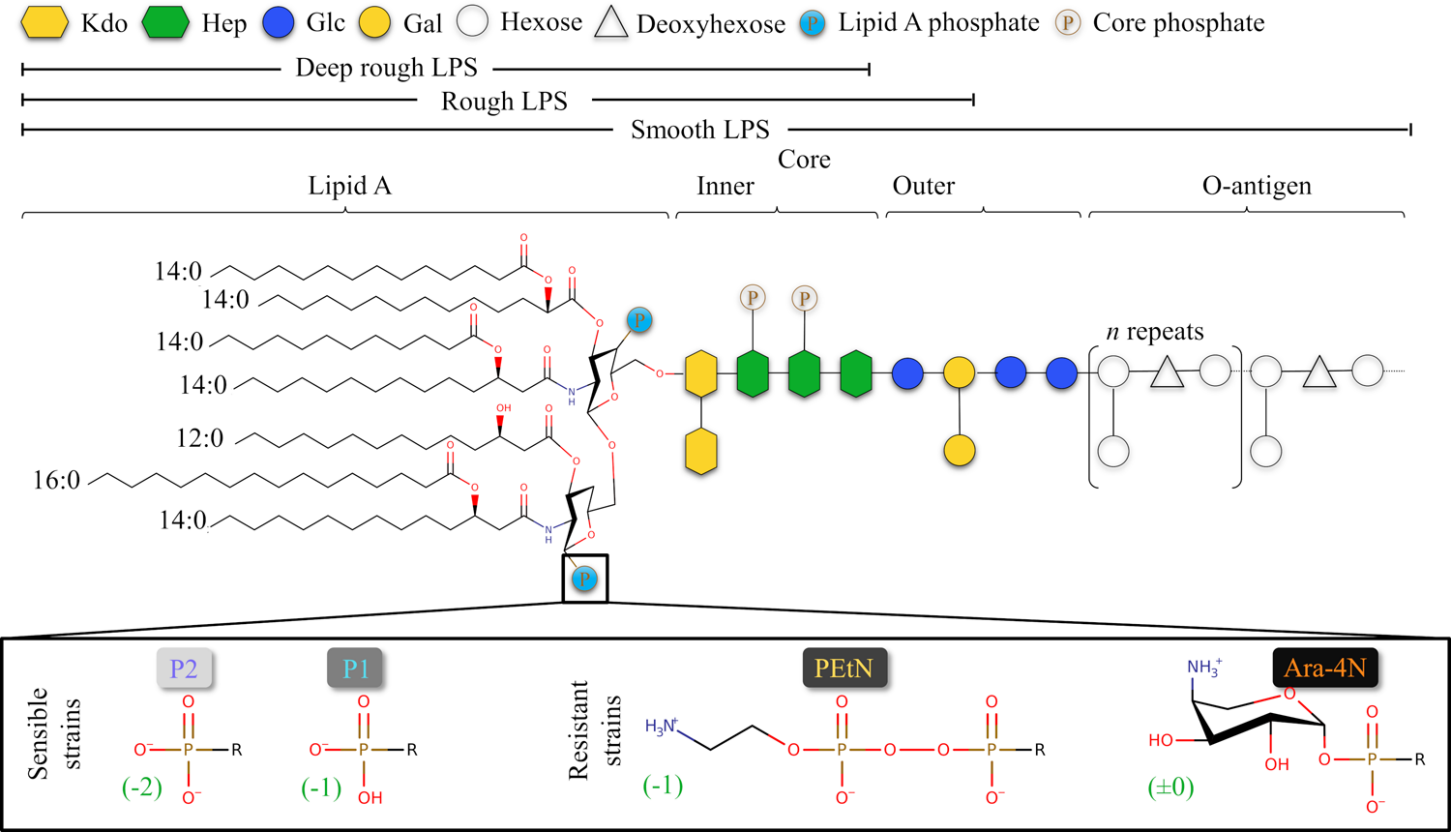
Chemical structures of the four models of LPS employed. The variation resides in the phosphate groups attached to lipid A (box at the bottom) with two models featuring phosphate groups that have different net charges (−2 or −1, depending on the protonation state). The other two models are decorated with PEtN or Ara-4N groups, which are associated with polymyxin resistance in bacterial strains.

We compared the effect of global stress in four different membrane models (Figure 2). The first model, P2, is the canonical model of a sensible strain of*Salmonella enterica*. A variant of that model, P1, assigns a net charge of −1 to the lipid A phosphate groups, as recommended by Rice et al.^47^ Next, two models are associated with mutations in the LPS held responsible to polymyxin resistance. The first of them, PEtN, binds a phosphatidylethanolamine group to the lipid A phosphate groups, while the second, Ara-4N, decorates these groups with 4-amino-4-deoxy-l-arabinose. This set of model membranes allows us to compare their ability to sustain stress and evaluate their impact on the content and distribution of divalent ions.

**Figure 3:**
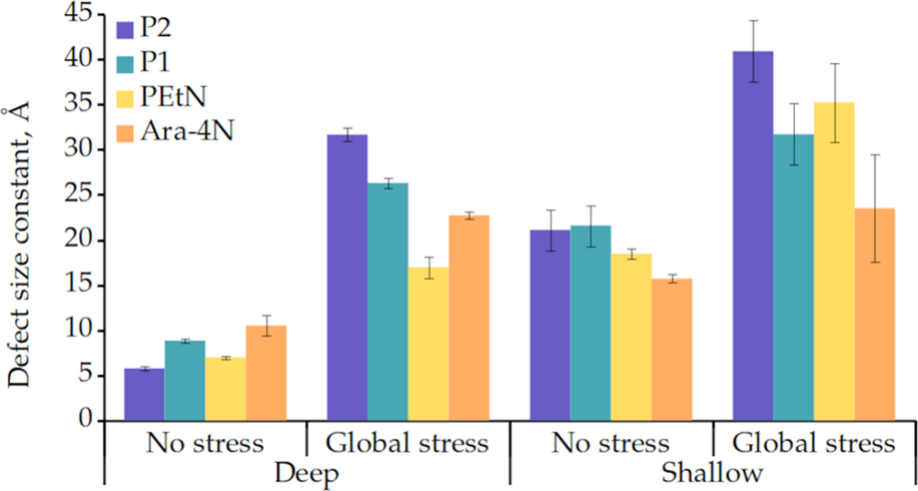
Packing defect size constants for equilibrated membranes and membranes under global stress.

In this regard, we compared the packing defect (Figure 3) induced by global stress to the four model membranes. We generated four replicas for each system, all of which were simulated until the variations in the density profiles of different relevant groups do not change with time (see Figures S2–S5). The removal of all calcium ions in the system drastically affects all model membranes that we studied. We observe a multifold increase of the deep packing defect size constants for all model membranes. Even in the case of PEtN, where the characteristic size of deep packing defect constant increases the least, it still grows from 0.70 ± 0.01 to 1.7 ± 0.4 nm. On the other hand, we see that P2 is the system that is most affected by such stress for both types of packing defects.

In the presence of calcium ions, the density of water molecules decreases more rapidly along the normal membrane, indicating that water penetrates the membrane less deeply. Susceptible strains (P2 and P1 models) display an increase in water in the surroundings of both layers of phosphorus/calcium ions, which fades when the ions are removed. Removal of divalent ions drives water much deeper, presumably through the cracks that are observed in the positions of LPS flipping (see Figure S7).

We calculated the distribution of the area per lipid using FatSlim.^48^ In the case of the system without stress (Figures S8 and S9), the LPS areas per lipid for both P2 and P1 share the same value (1.93 ± 0.01 nm^2^). We also notice that the largest area per lipid is for PEtN with a value of 2.11 ± 0.02 nm^2^, while Ara-4N displays a slightly lower area per lipid (2.07 ± 0.03 nm^2^). The removal of calcium ions dramatically widens the area per lipid distribution (Figure S8). Interestingly, the widening is most significant for P2, followed by P1, PEtN, and Ara-4N. However, it is not straightforward to estimate accurately the average area per lipid in systems that suffered such major stress because of the drastic widening of the distribution. Indeed, with the presence of cracks on the LPS monolayer, it is questionable whether the calculation of the area per lipid should exclude them, rendering the calculation substantially a harder task.

To complement the insight obtained with the area per lipid distribution, we also investigated the proportion of LPS that flipped through the simulation, as this observable shall be directly related to the change in LPS leaflet area. Table 1 shows the proportion of LPS that flipped from upper leaflet to lower leaflet.

**Table 1:** Percentage of LPS that Flipped toward the Lower Leaflet (POPE), and the Standard Error Associated^a^

system	proportion of flipped LPS	error estimate
P2	11%	2%
P1	9%	2%
PEtN	5%	1%
Ara-4N	1.3%	0.6%

a The proportion is computed from the density profile of the headgroup, as reported in the supplementary information (see Figures S10–S13).

### Modeling Local Ion Displacement

In the previous section, we addressed an extreme case in which the global stress associated with complete ion removal could be referred to as a chelator-like effect. On the other hand, that did not cover the local mechanism of ion displacement and its effects on membrane properties. In this section, we introduce a new collective variable that aims at describing the local polymyxin-induced ion displacement. Polymyxins, being polycationic lipopeptides, are believed to displace calcium or magnesium ions by means of repulsive electrostatic interactions. A straightforward way to model ion displacement would be to move those ions radially from the position of a membrane defect that is considered to be bound to one or several polymyxins. It should be noted that such a model does not require the presence of any polymyxins, focuses only on the influence of ion displacement, and thus does not involve direct interactions between polymyxins and the membrane. We propose a new collective variable ξ_dis_ that controls the number of calcium ions in a cylinder spanning transversally to the membrane. By isolating solely ion displacement, we aim to understand how such stress can affect membrane properties.

**Figure 4:**
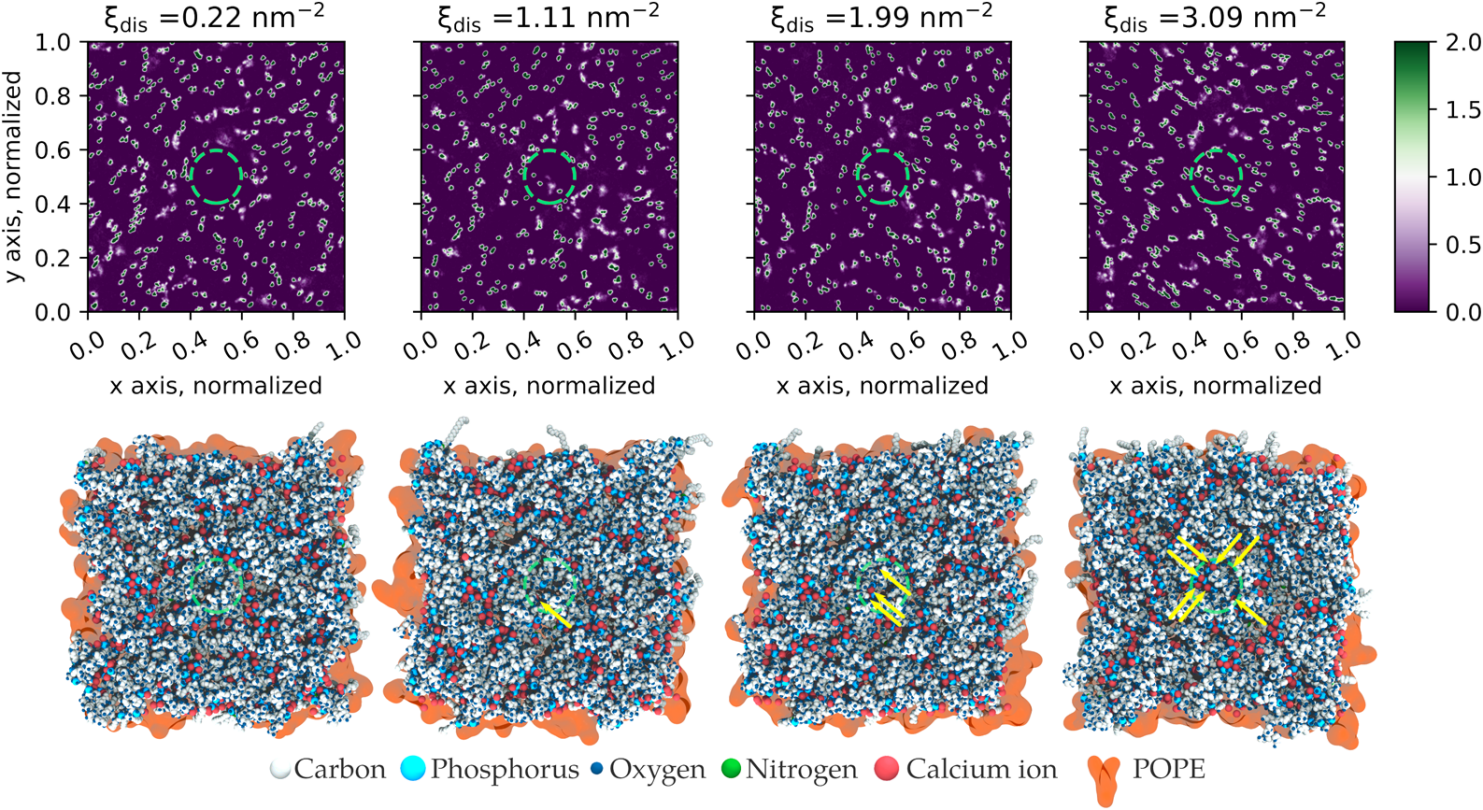
Upper panels show the density maps of calcium ions for a given value of the collective variable (see Methods for the CV description). Each map is associated with a representative snapshot of the system. Arrows point at calcium ions that are visible directly from the top view. The circles (in green) represent the cylinders where the biasing potential is applied. Number density values are in nm^–3^.

**Figure 5:**
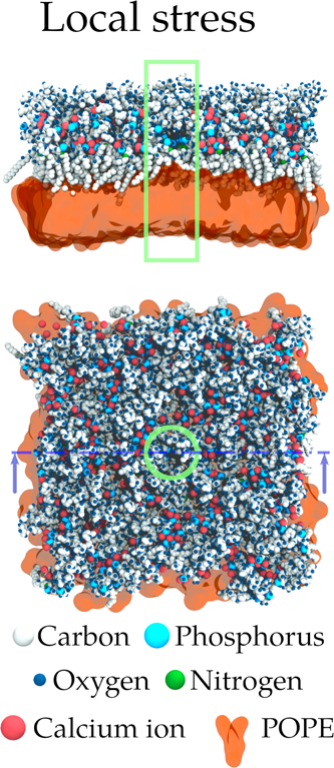
Representative snapshot from our simulations of one model of outer membrane of Gram-negative bacteria (system P2) under a local stress consisting of the local displacement of calcium ions away from a cylindrical volume centered in the simulation box (in green).

Figure 4 shows density maps and representative snapshots from MD simulations of the calcium ions in system P2, for different values of the collective variable where ξ_dis_ is expressed in number of ions per area unit, in the cylinder. This variable allows us to control the number of calcium ions in a local portion of the membrane (see also Movie S1 for the action of the collective variable on the calcium ions during a steered MD simulation). Interestingly, we do not see major membrane remodeling upon such local stress, as it is depicted in both Figures 4 and 5. Our results show a significant increase in the deep packing defect constant only in the case of P2 (see Figure S14).

**Figure 6:**
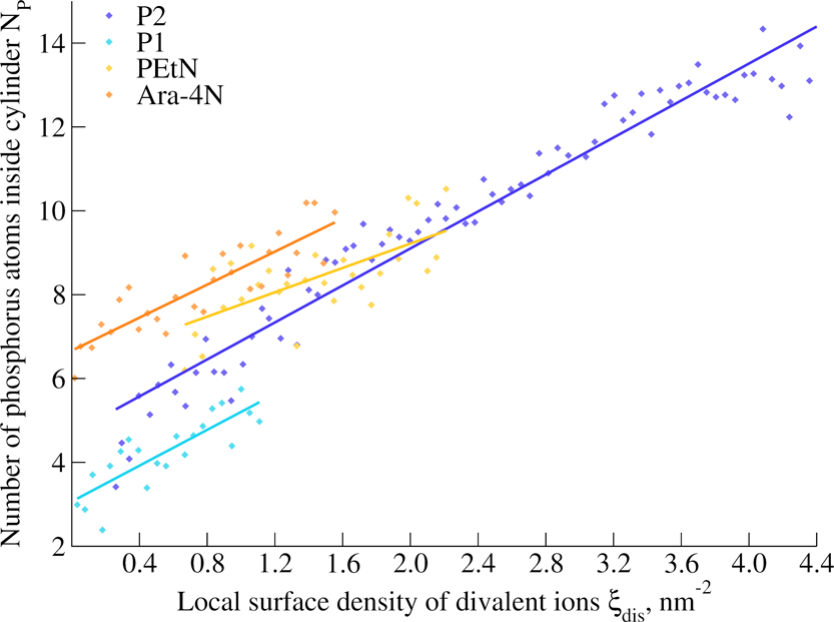
Variation of the number of phosphorus atoms in the cylinder as a function of the collective variable. Linear regressions are also plotted.

Figure 6 shows the variation in the number of phosphorus atoms in the cylinder with the values of the collective variable. P2, P1, and Ara-4N exhibit remarkably similar trends in the variation of the number of phosphates, which seems linearly correlated with the collective variable. The discrepancy observed for PEtN can be explained by the presence of one extra phosphorus atom in each of the lipid A phosphate groups. The observed co-motion of Ca^2+^ together with P does not mean that the whole LPS molecules are moving away from the cylinder. A small portion of the LPS, close to the phosphate groups, seems to leave the cylinder, while some LPS molecules rotate to ensure that the phosphorus atoms interacting with the displaced calcium ion(s) preserve this interaction (see also Movie S2 that shows the co-motion of phosphorus atoms along with biased calcium ions). This collective motion P–Ca^2+^ is a marked difference between local and global stress, which could play a role in the global conservation of membrane properties in the case of such local stress, by avoiding direct repulsion between unbridged phosphate groups.

Note that the local stress has small effects on the global membrane properties. For instance, there is no representative impact on the distance between phosphorus atoms of different LPS molecules (see Figure S15), nor any trends in the orientation of lipid A headgroups (see Figure S16). This stress also has little effect on membrane dynamic properties (see Figure S17). However, the local stress widened the area per lipid distribution, especially for P2, PEtN, and Ara-4N (Figure S9), while changes are not significant in the case of P1.

**Figure 7:**
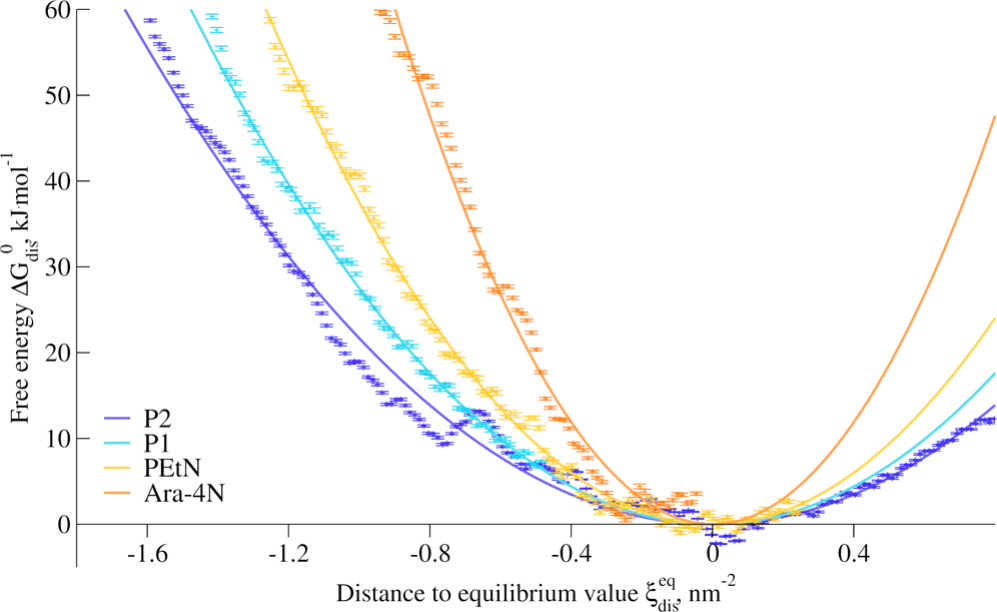
Free energy profiles of calcium displacement for various model membranes as a function of our collective variable. The profiles are shifted so that the minimum value of their quadratic fit is set to zero.

We computed the free energy profile of calcium displacement, along our collective variable (see Definition of the Collective Variable for further details); see Figure 7. To better estimate the surface density of calcium ions in each membrane in the absence of stress, we fitted a quadratic function that reads *k*_dis_(ξ_dis_ – ξ_dis_^eq^)^2^ + *k*_shift_ to the free energy profiles, in close vicinity to the equilibrium value. Here, the coefficient *k*_dis_ characterizes the stiffness of the quadratic function, *k*_shift_ accounts for the fact that free energy profiles are calculated only up to an arbitrary constant, and ξ_dis_^eq^ represents the position of the free energy minimum, corresponding to the number of calcium ions within the cylinder at membrane equilibrium. P2 has the highest minimum at ξ_dis_^eq,P2^ = 3.1 nm^–2^, while both P1 and Ara-4N share equilibrium value, at ξ_dis_^eq,P1^ = ξ_dis_^eq,Ara–4N^ = 1.6 nm^–2^. The equilibrium value for PEtN stands between those, at ξ_dis_^eq,PEtN^ = 2.0 nm^–2^. This points out that the net charge of LPS phosphate groups is not the only contribution to the divalent ion content in the membrane. We also estimated the average value ξ_dis_^unbiased^ for unbiased equilibrated membrane models; see Table 2. The differences observed in Table 2, between ξ_dis_^eq^ and ξ_dis_^unbiased^ suggest that the calcium density is not in equilibrium and is rather dictated by the membrane generator implemented in CHARMM-GUI, which determines the number of calcium ions inserted based on the charge of LPS molecules. While this approach gives estimated values close to our measurements, the equilibrium values slightly differ, showing the complexity of the interactions that could be partially screened and that could also depend on the presence of monovalent salts.

**Table 2:** Comparison of the Equilibrium Surface Density of Calcium Ions from Fitted Free Energy Minima and Unbiased Membrane Simulations

system	**ξ**_**dis**_^**eq**^, **nm**^**–2**^	**ξ**_**dis**_^**unbiased**^, **nm**^**–2**^
P2	3.1	2.6
P1	1.6	1.4
PEtN	2.0	1.9
Ara-4N	1.6	1.8

The free energy profiles allow us to better understand the contributions of the mutations affecting lipid A phosphate groups. The steeper the free energy profile is, the harder it is to create a calcium-depleted area in the membrane-bound ion network. Therefore, the quadratic fits of these free local profiles allow us to quantify the response of the system to the local stress. In this case, we can sort the different system based on the coefficient *k*_dis_, and see that *k*_dis_^P2^ = 22 ± 1 < *k*_dis_^P1^ = 28 ± 1 < *k*_dis_^PEtN^ = 38 ± 1 < *k*_dis_^Ara–4N^ = 75 ± 1 kJ mol^–1^ nm^–4^. Note that lower values of *k*_dis_ correlate with a higher propensity of flipping in the case of global stress.

### Local Stress and the Presence of Polymyxin

We simulated the P2-PME system, which contains one polymyxin E or colistin, restrained to the region of the cylinder. Since we are interested in the early stages of polymyxin modes of action, we decided to place the polymyxin molecule close to the top of the LPS layer. We want to address first whether polymyxin assists the formation of the calcium-depleted areas by making divalent ion displacement less energetically costly and second whether the presence of such an area assists polymyxin permeation through the outer membrane.

**Figure 8:**
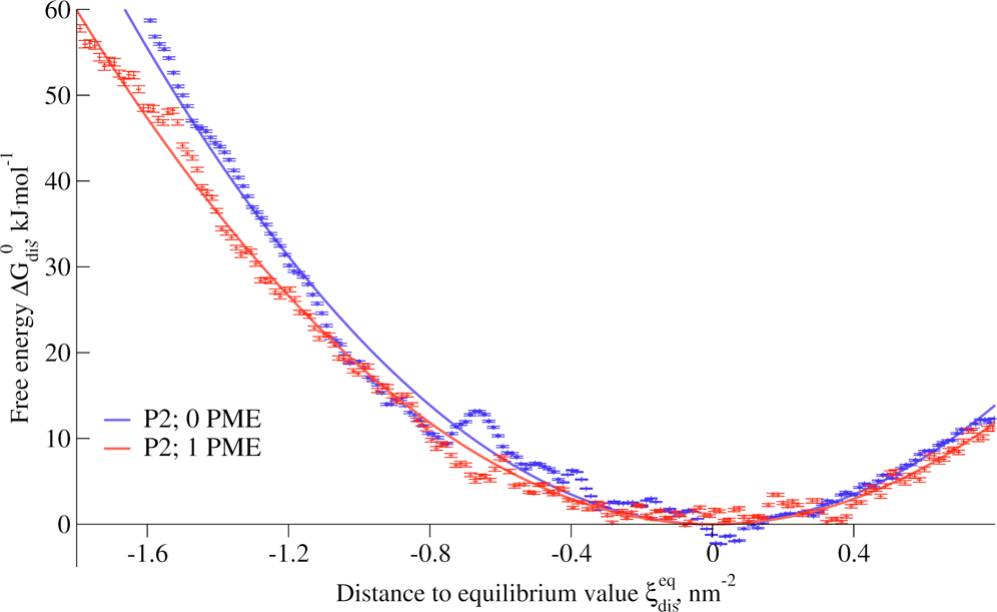
Free energy profile along our collective variable for the systems P2 and P2-PME, which is the model embedding one polymyxin. The profiles are shifted so that the minimum value of their quadratic fit is zero.

Figure 8 shows the free energy profile of calcium displacement for both P2 and P2-PME. There is no significant variation of the equilibrium value of the local surface density of divalent ions, as ξ_dis_^eq, P2^ = 3.11 nm^–2^, while ξ_dis_^eq, P2–PME^ = 3.08 nm^–2^, which can be explained by the presence of only a single polymyxin. However, polymyxin seems to decrease the stiffness of that free energy curve, reducing the cost to modify the local ion density with *k*_dis_^P2–PME^ = 19 ± 1 < *k*_dis_^P2^ = 22 ± 1 kJ mol^–1^ nm^–4^.

**Figure 9:**
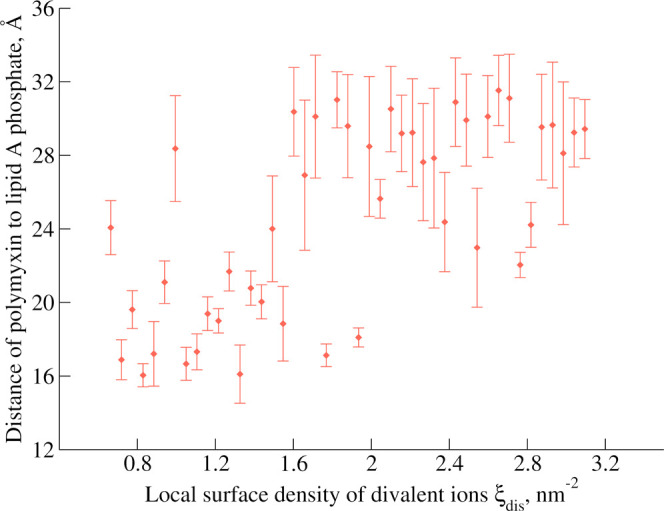
Evolution of the *z*-component of the distance between the center of geometry of polymyxin and that of lipid A phosphate groups as a function of the local surface density of divalent ions in the virtual cylinder, depicting that local stress affects the depth of insertion of polymyxin. Error estimate refers to the standard deviation over the last 100 ns of the simulation.

In the case of larger stress, i.e., lower density of divalent ions in the cylinder, the polymyxin molecule inserts deeper in the membrane (see Figure 9). We observe a threshold around ξ_dis_ ∼ 1.6 nm^–2^, below which polymyxin equilibrates ∼1.0–1.5 nm deeper in the LPS leaflet.

### Local Stress Effects in Coarse-Grained Simulations

**Figure 10:**
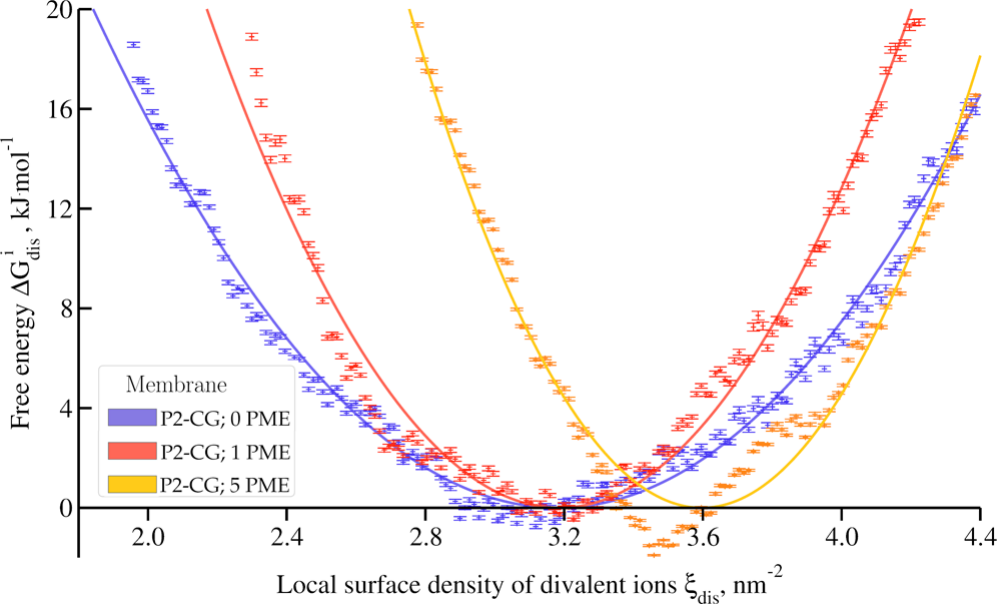
Free energy profile along our collective variable for P2-CG, P2-CG-PME, and P2-CG-5PME systems, using coarse-grained simulations embedding zero, one, and five polymyxin molecules, respectively.

Given that Martini 2 force field^45^ has been extensively used for studying interactions between polymyxins and OM models,^17,27,49,50^ we adapted our collective variable to this force field. At first, we computed (see Figure 10) the free energy profile with zero, one, or five polymyxin molecules (P2-CG, P2-CG-PME, and P2-CG-5PME). One can see that there is, once again, no significant variation of the equilibrium value of the local surface density of divalent ions, with ξ_dis_^eq,P2–CG^ = ξ_dis_^eq,andP2–CG–PME^ = 3.2 nm^–2^. However, the addition of five polymyxin molecules drives the equilibrium value to ξ_dis_^eq,P2–CG–5PME^ = 3.6 nm^–2^, which is a surprising result as one could expect the opposite trend, i.e., less ions in the presence of polymyxin. It is also surprising to see that, contrary to all-atom simulations, polymyxins seem to increase the energetic cost for calcium displacement, with *k*_dis_^P2–CG–5PME^ = 28 > *k*_dis_^P2–CG–PME^ = 19 > *k*_dis_^P2–CG^ = 11 kJ mol^–1^ nm^–4^.

**Figure 11:**
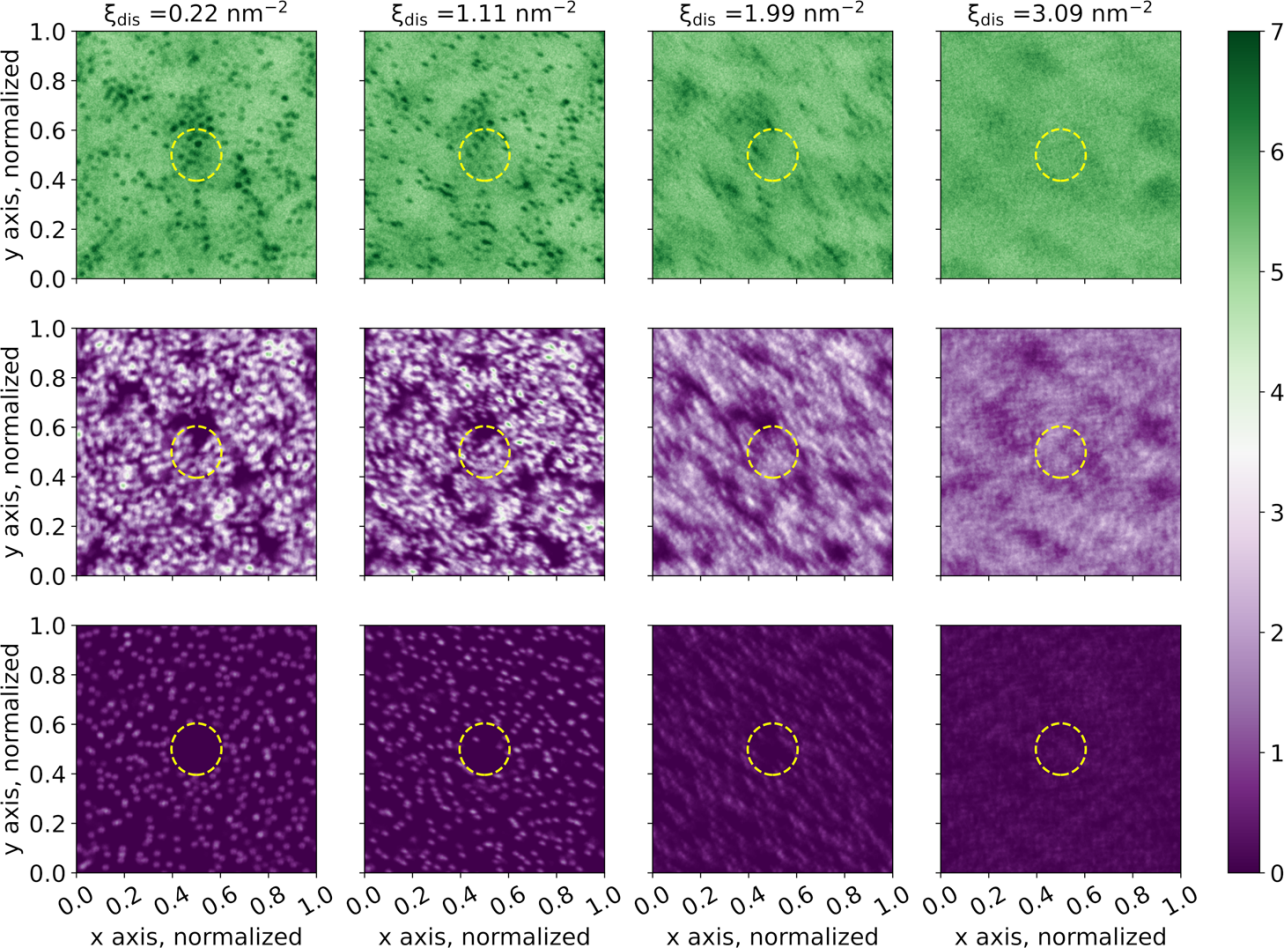
Time-averaged density maps of different groups in the case of P2-CG, for four values of the collective variable. The first row shows water molecules, the second row shows the LPS core, and the last row shows calcium ions. Number density values are in nm^–3^. The yellow circles indicate the position of the virtual cylinder in which the biasing potential is applied.

To explain the role of polymyxins in the displacement of divalent ions, we investigated the time-averaged density maps of different groups in the system, namely, water, LPS core, and calcium ions (see Figure 11). One can see that calcium beads, LPS core beads, and water present in the surroundings of the lipid A headgroup and LPS core are *freezing* while we decrease the density in calcium in the cylinder. Sharp density changes observed for low calcium concentrations indicate that, during the analyzed time (last 1000 ns), the groups mentioned above did not exhibit normal thermal fluctuations. Freezing occurred in all coarse-grained-simulations, with and without the polymyxin molecule. It is more pronounced for lower values of collective variable, i.e., for lower ion densities in the cylinder.

**Figure 12:**
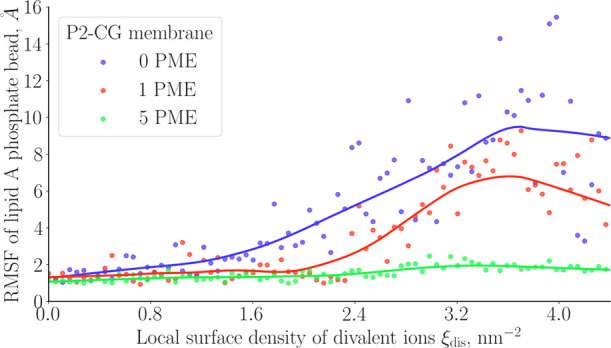
Variation of the RMSF of lipid A phosphate beads as a function of the collective variable. Plain lines are data smoothing based on local regression smoothing (LOESS).

For better understanding, we plotted the time-averaged root mean square fluctuations (RMSFs) of the phosphate beads that are highly affected by the freezing, as a function of the collective variable, and for different numbers of polymyxin molecules in the simulation box (see Figure 12). Such an evaluation of the RMSF was already made to assess the relative flexibility of a given region of LPS.^7^ We see that the more polymyxin molecules are present in the cylinder, the more phosphate beads exhibit low RMSF for higher values of the CV. We also noticed that the RMSF values drastically drop for smaller values of the collective variable, i.e., for low surface densities in divalent ions in the calcium-depleted site.

## Discussion

### The Complete Removal of Divalent Ions Dramatically Affects Structural Properties of the OM

The complete loss of the calcium ions present in our model membranes induces repulsive interactions between phosphate groups of lipid A and the LPS core, resulting in a major and rapid increase in the upper leaflet area. The lower leaflet cannot adapt to such global stress, leading to the appearance of transient lipid islands in the lower leaflet, which do not sustain a continuous monolayer. The remodeling of the lower leaflet further drives some LPS to flip toward the POPE layer, which is visible in Figure 1. Even though the membrane remains a bilayer in our simulations, this important remodeling could drastically affect the permeability of that membrane.

This global membrane remodeling is in agreement with works showing the chelation of membrane-bound calcium ions by ethylenediaminetetraacetic acid (EDTA).^51−54^ Clifton and co-workers also observed membrane LPS flipping into the inner leaflet in their simulations in line with their experiments in which they wash out calcium ions with EDTA.^55^ However, it is important to consider that while comparing experimental studies using EDTA with MD simulations, where divalent ions are simply removed, can provide valuable insights, the simulations did not include the binding of EDTA into the bilayer, which may affect the results.^56^ In this regard, it is interesting to note that Fu and co-workers^57^ reported that higher concentrations in polymyxin may assist lipid scrambling. Additionally, we are aware that the size of the membrane patch could impact some of our results, which is why we used system size similar to previous studies,^27,58−60^ where the number of LPS molecules per leaflet ranges from 8 to 64, while our systems contain 79 LPS molecules.

Our results indicate that P1 and P2 are very sensitive to global stress in comparison to PEtn or Ara-4N, despite all of them having the same number of acyl chains. This observation tends to confirm the conclusions indicating that hepta-acylation of LPS is not a marker of polymyxin resistance.^61−63^ In addition, Santos and co-workers showed, with MD simulations, that both penta- and hexa-acylated lipid A membranes are susceptible to polymyxin.^40^ Together, the data indicate that phosphate group modifications may affect the susceptibility to polymyxin more than the number of acyl chains.

In the simulations depicted in Figure 1, the removal of the divalent ions did not cause a major buckling of the membrane, nor did we see an increase in the separation between leaflets as it was reported before.^27^ We confirmed the absence of curvature with our analyses using the SuaAVE package.^46^ The range of values that we measured for the position of the peak in the distribution of the surface angle, which is well below 10°, indicates that our model membranes are flat, following the conclusions by Santos and co-workers.^40,46^ We also note that there is no systematic shift of this peak between the membranes simulated in absence of stress and the membranes in the presence of global stress. Although this analysis shows that our membranes stay planar under stress, we observe a broadening of the distribution of surface angles, indicating an increase in the amplitude of membrane fluctuations under global stress. It is worth noting that the size of our simulated membranes, which is comparable to those used in similar studies, can influence fluctuations and undulations, as larger systems are better suited to capture long-wavelength deformations. Additionally, the choice of force field is a major factor affecting these observations. By comparison with the simulations relying on the GROMOS force field, detailed by Santos and co-workers,^40^ we observe a more marginal broadening of this distribution, indicating that the CHARMM36 force field drives LPS-containing membranes to be much more rigid than the GROMOS force field. Similarly, we do not observe the loss of the characteristic lamellar structure toward an amorphous one. However, it has already been discussed that these changes in membrane structure are largely force field dependent.^47,49,60^ It was reported that CHARMM36 and Martini force fields do not show such drastic changes upon divalent ion removal from the membrane, while this was observed with the Gromos^60^ force field. Interestingly, the GLYCAM force field was also shown to not produce stable bilayers when all cationic salts are monovalent potassium ions,^47^ which may come from issues with the representation of intermolecular interactions of saccharides that tend to be overestimated.^64^

It has been commonly reported that the displacement or the (partial) removal of calcium ions in the OMs is a key mechanism in the action of polymyxins.^28,65,66^ However, Manioglu and co-workers showed that polymyxins, upon binding to OM, form crystalline structures that contribute to their antibacterial activity.^67^ Furthermore, the formation of these structures is dependent on the presence of divalent ions, indicating a mechanism more complex than previously thought. While it is established that polymyxins’ interactions with OM divalent ions are crucial for permeabilizing the OM, it remains an open question whether polymyxins directly induce membrane rupture or if the displacement of divalent ions primarily facilitates polymyxin internalization in the bacterium. Moreover, polymyxin nonapeptides, which lack the fatty acid moiety, are characterized by significantly reduced antimicrobial activity.^25^ The optimal length of that fatty acid was shown to lie between seven and nine carbons long, leaving lipopeptides with different chain lengths to display reduced antimicrobial activity.^19,21^ The complexity of this picture has increased as recent studies have begun to address the roles of OM proteins in interactions with polymyxins,^68^ showing that the mode of action of polymyxins is not limited to the sole divalent ion displacement.

Our results, i.e., lipid packing defect, widening of the area per lipid distribution, and the proportion of LPS flipping, support that P2 is more sensible to divalent ion removal than polymyxin-resistant models. The P1 model, in which the net charge of lipid A phosphate groups is set to −1, which represents a fully protonated state of lipid A phosphate groups,^47^ seems to be an intermediate between P2 and polymyxin-resistant strains. The change in the net charge of lipid A phosphate thus should not be considered alone, as the type of chemical group that decorates these phosphate groups also affects how the membrane reacts to global calcium removal.

We noticed that divalent ion removal increases water penetration in the membrane. It is interesting to note that earlier studies^13,60^ have pointed out that the presence of divalent cations, either Mg^2+^ or Ca^2+^, diminishes water penetration through LPS layers, although Mg^2+^ does not act as much as Ca^2+^. This increase correlates with the presence of larger shallow and deep packing defects in the membrane and with the increase in the area per lipid of LPS (Figures S8 and S9). The distinct behavior of Mg^2+^ and Ca^2+^ ions in the bacterial OMs can be attributed to their intrinsic differences. Although both ions share the same charge and electronic configuration in their outermost s-orbital, their physicochemical properties differ significantly. Ca^2+^ has a larger atomic radius, but Mg^2+^ exhibits a higher hydration radius and greater hydration energy.^69^ The flexibility of Ca^2+^’s solvation shell could allow it to form stronger salt bridges with LPS molecules, resulting in tighter packing and decreased water penetration into LPS-containing membranes.^70^ In contrast, Mg^2+^’s larger hydration shell may limit its ability to form such bridges, leading to comparatively looser packing and slightly increased water penetration. This hypothesis aligns with X-ray reflectivity and diffraction studies showing lower compressibility for Ca^2+^-bound LPS layers compared to Mg^2+^-bound ones.^47^

The reaction of polymyxin-sensitive models, P2 and P1, to such global stress is in line with the conclusions made by Lam and co-workers.^13^ In their theoretical work, the authors quantify the electrostatic modifications of the LPS layer using a very coarse-grained model, demonstrating that the presence of divalent ions (Mg^2+^) induces a negative lateral pressure, which tightens the LPS layer, whereas monovalent ions cause a positive lateral pressure, leading to the swelling of lipid headgroups. In other words, the presence of magnesium ions turns the repulsive interactions between LPS molecules into attractive interactions.

Rice and co-workers made a seminal work regarding the influence of both ion content and phosphate groups in OM models, relying on the CHARMM36 force field.^47^ They showed that modifications of the lipid A phosphate groups that are linked to resistance mechanisms to polymyxins make the model membranes much less sensitive to the type of ion (monovalent or divalent) used in their simulations. These conclusions are in line with our observations based on the lipid packing defect and the area per lipid.

### Local Ion Displacement Is More Energetically Costly in Polymyxin-Resistant Membranes

The collective variable we introduce in this work allows us to address how membrane-bound divalent ion displacement is affected by the LPS content of the membrane models. Jefferies et al.^50^ reported that polymyxins did not induce major changes of the physical properties of the model membranes. Fu and co-workers^41^ also pointed that changes of the lipid packing defect are not significant for polymyxin/lipid ratios under 2%. Hence, we expected that a local stress that mimics only a portion of polymyxin interactions will not have significant effects on membrane properties but that ion displacement is likely to be affected by the composition of membranes.

Santos et al., by measuring the calcium diffusion coefficient in their MD simulations, demonstrate that ion mobility is less affected in polymyxin-resistant model membranes, strongly corroborating our conclusions from free energy calculations.^40^ Additionally, they indicate that ion displacement upon polymyxin exposure primarily occurs along the membrane surface, which our simulations support by showing minimal desorption—i.e., one to two events when displacing all ions from the cylindrical area where we apply the biasing potential—of calcium ions from the membrane (see Movie S1).

It is important to point out that the collective motion calcium–phosphorus might depend on the precise LPS parametrization. Indeed, Rice et al.^47^ reported that the CHARMM force field tends to overestimate cation binding. They hypothesized that this comes partly from the −2 net charge attributed to the phosphate groups of lipid A. Based on this report, we also simulated system P1 with −1 net charge for the lipid A phosphate groups, which keeps showing that collective motion.

### Polymyxins Facilitate Ion Displacement and Ion Displacement Facilitates Polymyxin Adsorption

Our results support that an adsorbed polymyxin molecule can increase the mobility of membrane-bound divalent ions. That observation is in agreement with the results obtained by Santos and co-workers,^40^ where they led united atom simulations showing that the presence of polymyxin B increases the calcium diffusion coefficient by about a twofold rate or higher when the drug is adsorbed on the membrane surface.

The increased ion mobility suggests that polymyxins contribute to ion displacement, even in the early stages of polymyxin–membrane interaction, when the drug is merely adsorbed on the membrane. This aligns with observations by Berglund et al., who reported intermittent but no long-term displacement of membrane-bound ions upon polymyxin B_1_ interaction.^27^ Our free energy profiles show that while the equilibrium value ξ_dis_^eq,P2^ is marginally affected by polymyxin presence, the decrease of *k*_dis_ indicates enhanced calcium ion mobility. Additionally, Jiang et al.^43^ report coarse-grained MD simulations showing that divalent ion displacement remains energetically unfavorable in the presence of polymyxin.

It is not clear how polymyxins reach a state upon which they displace membrane-bound divalent cations. Polymyxin insertion remained shallow throughout our simulations, with polymyxins entering no farther than the outer core of LPS. This is in line with the previously reported presence of multiple energy barriers for deeper insertion of polymyxin or other relevant drugs.^50,57,71−73^ Hence, it is an open question whether the presence of a calcium-depleted area should pre-exist to promote this (partial) translocation of polymyxins or if a concentration threshold should be reached on the membrane to facilitate this permeation, questioning the initial steps of the self-promoted model presented by Hancock and Chapple.^74^ We observe that the presence of an area with a disrupted distribution of divalent ions favors the insertion of a polymyxin molecule. Polymyxin-induced calcium depletions could thus be self-fed and drive more polymyxin molecules to insert. This observation aligns with numerous studies indicating that polymyxins can penetrate deeply into the hydrophobic region of the OM and even reach the IM.^21,23,75^ Particularly, there is experimental evidence that polymyxins accumulate in the KDO/headgroup region.^29^ However, one cannot assume that polymyxin partial translocation is a necessary step in its bactericidal activity, as there is evidence showing that polymyxin internalization across the OM is not necessary to alter OM properties and damage the cell envelope.^39,76^

### Coarse-Grained Simulations and Local Stress

The results from our coarse-grained MD simulations using the Martini 2 force field^17^ present a notable divergence from the all-atom simulations when analyzing the behavior of divalent ions in the OM. While atomistic simulations showed a widened free energy profile along the collective variable in the presence of polymyxins, indicative of increased ion mobility, the coarse-grained simulations exhibited a stiffer free energy profile, suggesting reduced ion mobility, in the presence of polymyxins. This discrepancy can be attributed to the *freezing* effect observed in the coarse-grained simulations, where divalent ions induce localized immobilization of adjacent beads, including phosphate and water molecules.

A recent experimental study highlighted the formation of crystalline structures in LPS upon interaction with polymyxins, providing context for our observations.^67^ It raises the critical question of whether this freezing is a force-field-linked artifact, especially since such freezing was not observed in our atomistic simulations. The free energy profiles, which differ significantly between the simulations with and without polymyxin, underscore the effect of this freezing phenomenon.

The parametrization of divalent ions within the Martini 2 force field might be a major contribution to the observed differences with our atomistic simulations. Previous work by Jefferies et al.^50^ reported that polymyxin B_1_ could induce local transitions, driving model membranes toward glass-like properties in simulations using Martini 2. Our observations align with these findings, as the collective variable displacing calcium ions—mimicking polymyxin action—drives the membrane to a frozen state. Increasing the polymyxin concentration accelerates this transition, corroborating earlier reports. The freezing events are present even in the early steps of our coarse-grained simulations, and thus, the limited time scales in all-atom simulation are likely not the issue.

## Conclusions

Over the years, the global picture of polymyxin-mediated bacterial death has been shown to be intrinsically linked to actions at the OM level. Despite new evidence highlighting the role of porins in the mode of action of polymyxins, direct interactions with LPS remain critical, as they solely explain the mechanisms of some of the polymyxin-resistant mutations. Our work demonstrates that both structural modifications of the OM and dynamical properties of the divalent ions are affected by stresses mimicking parts of polymyxin interactions with the OM.

We showed that polymyxin-sensitive model membranes P2 and P1 are significantly more affected by ion removal than polymyxin-resistant ones, with Ara-4N being the least affected and PEtN being intermediate. Specifically, P2 exhibits the most significant widening of the LPS area per lipid, the largest increase in the lipid packing defect constant, and the highest proportion of LPS flipping from the outer leaflet to the inner leaflet.

By introducing a new collective variable to monitor the local process of ion displacement in the membrane, we demonstrated that ion mobility is influenced by the type of LPS present. Notably, P2 is more affected than P1, which, in turn, is more affected than PEtN, with Ara-4N being the least affected. The addition of a single polymyxin molecule further increased the ion mobility in the P2 system. Concurrently, we showed that defects in the membrane-bound divalent ion network facilitate the internalization of adsorbed polymyxin, supporting the self-promoted uptake model proposed by Hancock.

Our results also indicate that the force field employed in the simulations plays a crucial role in the observed effects of ion displacement on membrane properties. The use of Martini parametrization leads to the freezing of the entire region surrounding the LPS phosphate groups in our coarse-grained systems, which could correspond to experimental observations of LPS crystallization and findings from other studies using this force field. Nevertheless, this transition was not captured in our atomistic simulations and heavily relies on electrostatic interactions with divalent ions.

## Methods

### Models of the All-Atom and Coarse-Grained Topologies of Colistin

The initial structure of colistin and its SMILES description was taken from the ChEMBL database,^77^ and it was later translated with OpenBabel^78^ into mol2 format and hydrogens were added accordingly to pH 7.4. The obtained structure was loaded into CHARMM-GUI^79^ Ligand Modeler.^80^ Ligand Modeler produced topology files and all necessary parameters compatible with the used CHARMM36 force field for GROMACS. These parameters were closely checked afterward for correspondence with existing parametrizations of amino acids present in colistin to ensure that Modeler was able to grasp structural properties of the molecule.

To assess the possible inaccuracies in our topology, we use the same method described by Santos et al.^40^ to compute the vicinal coupling constants for polymyxin E, in order to explore the conformational dynamics of that molecule in solution and upon binding to LPS membranes (see Table 3). When comparing with the experimental data, we find a root mean square deviation (RMSD) of 0.5 that is lower than the values obtained by Santos and co-workers.^40^

**Table 3:** Vicinal Coupling Constants for Polymyxin E as Measured by NMR^81^ and Compared from MD Trajectories^a^

residue	experimental vicinal coupling constant	computed vicinal coupling constant
DAB1	7.16	7.9 ± 0.5
THR2	6.36	6.6 ± 0.4
DAB3	7.40	7.9 ± 0.5
DAB4	7.34	6.8 ± 0.5
DAB5	8.32	8.2 ± 0.9
R-6	6.01	6.6 ± 0.9
R-7	5.93	6.6 ± 0.5
DAB8	7.73	7.3 ± 0.9
DAB9	7.43	8.0 ± 0.6
THR10	7.41	7.0 ± 0.5
RMSD	**0.5**	

a The standard errors over the computed values are indicated. The root mean square deviation (RMSD) between experimental and computed values is indicated.

To create coarse-grained topology, PyCGTOOL^82^ was used. The all-atom modeled molecule of colistin was solvated, and counterions were added to neutralize the charge and ensure physiological salt concentrations and run for 100 ns to produce a simulation trajectory, which was used as an input for PyCGTOOL. Mapping design was created in a similar way as it was done for polymyxin by Fu et al.^57^

### Modeling the Lipid Matrix of the Outer Membrane of Gram-Negative Bacteria

Lipopolysaccharides consist of numerous fatty acid chains, typically ranging from 4 to 7. The higher number of tails in lipid A than in most phospholipids confers a higher contact surface between LPS and leads to tighter tail packing.^83^ Both properties contribute to the high stability and low fluidity of OM and to antibiotic resistance. Above lipid A, core oligosaccharides greatly participate in the lamellar structure of the OM. This contribution is attributed to negatively charged moieties, i.e., phosphoryl and carboxyl groups, bridged between LPS by a network of divalent ions (Ca^2+^ and Mg^2+^), which further enhances membrane rigidity and stability.^7,10,84,85^ The O-antigen, a polymeric chain of saccharides of variable lengths,^16,86^ is covalently bound to the core. Together, they constitute the hydrophilic layer of the OM that favorably interacts with the surrounding medium. In Gram-negative bacteria, LPS exhibit diversity in the number of fatty acid chains, their lengths, and the composition of saccharides in the core and the O-antigen. This array of variations defines distinct chemotypes.

Few numerical studies,^17,49^ especially at the atomic scale,^7,60,87^ utilize smooth LPS, i.e., LPS molecules that include the O-antigen. The length of the O-antigen significantly increases the size of the systems, adding substantial challenges to the already long sampling times required for OM models due to slow motions of the lipopolysaccharides. Hence, many OM models rely on truncated versions of smooth LPS that conserve the essential parts of LPS that interact with many antibiotic families. A few models represent the OM with LPS modeled solely as lipid A.^27,40,88−91^ However, it is common to use deep rough LPS (Re-LPS),^27,40,41,50,72,92,93^ which consists of lipid A bound to the Kdo units of the inner core and which represents the shortest core configuration able to maintain bacterial membrane integrity. These mutants lack core phosphate groups believed to play crucial roles in membrane structure and in its interaction with polymyxins. Therefore, rough LPS should be considered in models focusing on such interactions.^94^ Rc-LPS^47,83^ and Ra-LPS^43^ are two commonly described rough LPS, with Rc-LPS being slightly shorter than Ra-LPS. Studies have shown that these additional saccharides influence membrane properties, including the ordering of the phospholipid leaflet,^95^ which motivated our choice to rely on Ra-LPS for the outer leaflet of our OM models.

In our work, we focus on models of the OM of *Salmonella enterica*, a pathogen known for causing salmonellosis. *S. enterica* was also shown^96^ to develop resistance against polymyxins relying on modifications of the lipid A phosphate groups that are present in the CHARMM-GUI membrane builder^79,97^ online tool, allowing a direct comparison between polymyxin-sensitive and polymyxin-resistant models.

All systems in our study share the same number of acyl chains, with differences between LPS molecules arising solely from variations in the lipid A phosphate groups. The selection of a specific number of acyl chains is not straightforward. Polymyxin-induced addition of PEtn or Ara-4n groups occurs systematically through activation of pmrA/pmrB via the PhoP/PhoQ system, leading, in *S. enterica*, to concurrent LPS modification with a palmitate by PagP.^98−100^ Consequently, polymyxin-resistant membranes exhibit hepta-acylated LPS, exceeding the six acyl chains typically found in many wild-type strains of *S. enterica*. It is noteworthy that wild-type *S. enterica* and *Escherichia coli* strains can exhibit hepta-acylated lipid A without any lipid A phosphate modifications.^101−103^ This phenomenon may also occur for pmrA- mutants.^104^ Moreover, hepta-acylation of LPS may not be a marker of polymyxin resistance.^61−63^ These observations drove us to use seven fatty acid chains in both polymyxin-sensitive and polymyxin-resistant models.

### All-Atom Models of the OM of Gram-Negative Bacteria

Four models of LPS molecules were used to create four different model membranes (Figure 2). Two models, P2 and P1, represent membranes of nonresistant strains of *S. enterica*, while the two other models represent membranes with PEtN or Ara-4N groups decorating both lipid A phosphate groups. Each membrane is made of 79 LPS molecules in the upper leaflet and 255 POPE molecules in the lower leaflet, so that the area of each leaflet matches one another, based on the predictions provided in the CHARMM-GUI membrane builder. The number of divalent ions (calcium ions) embedded in the membrane is generated by CHARMM-GUI. Their number is based on the total net charge of the LPS molecules, and they act as counterions to the LPS molecules. One extra system, embedding one polymyxin, was also modeled using the P2 system. All-atom MD simulations comprise (1) an equilibration step, based on the steps proposed by CHARMM-GUI for membrane equilibration; (2) an extra equilibration step in case polymyxin is added to the system, (3) a set of four replicas of simulations with calcium ion removal; (4) a steered MD simulation pulling along the collective variable, which uses the last frame of the equilibration run; and (5) an umbrella procedure that was used to calculate the free energy profiles using the weighted histogram analysis method^105^ (WHAM).

#### Equilibrium Step

To check that our model membranes were at equilibrium, we monitored the changes in box size as well as the temperature and continued our simulation for more than 100 ns, reaching 300 ns in total.

#### Extra Equilibration Step in Systems Containing Polymyxin

Simulations with single polymyxin were prepared from the frame corresponding to a simulation time of 100 ns, using the umbrella window for systems without polymyxin, corresponding to the highest value of the CV. The system was equilibrated with that single polymyxin, with addition of extra counterions to neutralize it, for an extra 100 ns. Then, the same procedures of the steered MD simulation and umbrella sampling were applied.

In simulations that involve polymyxin, we decided to mimic the hypothesized aggregation process that supposedly occurs at the position of the already calcium-depleted area. To do so, we limited polymyxin only in a concentric cylinder to that of the CV, defined in the same way, but with *r*_0_ = 3.0 nm, and an additional upper wall potential distant by 6.0 nm from the plane of the membrane center of geometry that is defined by the lipid tails. That latter potential was present to avoid polymyxin leaving the LPS-containing leaflet during simulations while allowing polymyxin molecule(s) to rearrange structurally. Both extra potentials were set with a weak force constant κ = 100 kJ mol^–1^ atom^–1^.

#### Ion Removal Simulations

Each simulation involving ion removal was run for 300 ns. Each simulation was started from a random frame from the last 100 ns of the equilibrated simulations. Solvent as well as all membrane-bound ions were removed, and the system was resolvated with as many chloride ions as in the original system and neutralized with addition of sodium ions. The box size was increased to 20 nm along the *z*-axis before equilibration, which corresponds to an increase in the number of water molecules by 45 to 65%, depending on the system. This was done to ensure that fluctuations of the membrane while out-of-equilibrium will not drive the system to self-interact. For these systems, we also monitored the changes in the density profiles of representative groups and waited until the two last 25 ns blocks of simulations showed no variations that would be bigger than the statistical fluctuations expected in our systems. Hence, we could consider the last two blocks of our whole trajectory, i.e., the last 50 ns, to be close to equilibrium. The last 25 ns was used for analysis. Four independent replicas were always used to obtain the averages.

#### Steered MD Simulations

Our steered MD simulations consisted of a run with a rate of ∼1.1 ions/nm^2^/ns or 5 ions/ns and a force constant κ = 2000 kJ mol^–1^ ion^–1^. We monitored the reaction of the system to the applied bias by plotting the value of the measured CV versus the value of the applied bias (Figure S18). This setting results in a linear response for which the shift between the measured value and the applied bias is always very small, the slope of the linear regression being 1.02 for a correlation coefficient of 0.997.

#### 5 Umbrella Sampling Procedure

The umbrella procedure was carried out by applying a biasing potential with a force constant κ = 500 kJ mol^–1^ ion^–1^. Figure S19 shows that the histograms had enough overlaps. We also checked the convergence of the free energy profiles by analyzing 25 ns blocks of our all-atom simulations (Figure S20) and 200 ns blocks of our coarse-grained simulations (Figure S21). Simulation times, number of windows, and equilibration times are reported in Table 4. Errors on the free energy calculations were calculated by means of a bootstrapping method using 200 bootstraps.

**Table 4:** Simulation Times for the Production Runs of the Different Systems^a^

system	equilibration	steered MD	umbrella sampling	number of windows
P2	300 ns	>75 ns	300 ns/window	77
P1	300 ns	>75 ns	200 ns/window	21
PEtN	300 ns	>75 ns	200 ns/window	29
Ara-4N	300 ns	>75 ns	200 ns/window	29
P2-PME	100 ns	>75 ns	200 ns/window	68

a Note that the equilibration time with the polymyxin molecule (P2-PME system) started after inserting the polymyxin on the equilibrated P2 membrane.

### Definition of the Collective Variable

The collective variable is defined as the number of calcium ions in a cylinder of *r*_0_ = 1.2 nm of radius centered in the center of geometry of the membrane without periodic images. The radius of that cylinder was chosen to encompass the characteristic size of an aggregate of polymyxins, noting that a growing number of observations drive to the conclusion that polymyxins do not access the membrane surface individually.^33,27,41^ Soft boundary conditions defined by a switching function such that  where *n* = 14 were applied. As visible in Movie S1, most degrees of freedom of the calcium ions are not affected by our CV, resulting in ions that generally move radially due to the lamellar substructure of the LPS but also to few events of ions exiting the membranes (in orange in the movie).

Our collective variable is defined as a number of atoms/beads (calcium ions) present in a cylindrical volume and implemented with PLUMED library.^106^ Values are then converted, in order to express our collective variable as a surface density, by straightforwardly dividing the values of the collective variable by the surface of the cylinder: i.e., by 1.2^2^π.

Unless stated otherwise, our collective variable was ported to a coarse-grained system without modifications.

### Parameters Used with Atomistic Simulations

All atomistic simulations that required an equilibration procedure used a set of energy minimizations, canonical ensemble simulations, and isobaric–isothermal ensemble. These simulations gradually alleviate a set of position restraints that affects the lipids, while increasing the time step. The NVT and NpT equilibration simulations are relying on the Berendsen algorithm for the thermostat and/or the barostat for the first short steps, according to the protocol provided by CHARMM-GUI,^97^ before switching to the Nosé–Hoover algorithm for the thermostat and Parrinello–Rahman algorithm for the barostat, using a coupling constant of 1.0 and 5.0 ps, respectively. Temperature coupling was made separately for the membrane and the solvent. The simulations are held at 310 K and 1.0 bar. Dispersion interactions are treated with a cutoff set at 1.2 nm where forces are smoothly switched to 0 from 1.0 nm. Electrostatic interactions are treated using the fast smooth particle-mesh Ewald^107^ (SPME) algorithm, implemented in Gromacs package version 2022.3^108^ that was used all along this work and patched with the PLUMED library^106^ version 2.8.1.^109^ All simulations used for producing the data that was later analyzed used a 2 fs time step with all hydrogen bonds treated as constraints using the LINCS algorithm.^110^

### Coarse-Grained Models of the OM of Gram-Negative Bacteria

Coarse-grained models all rely on the Martini equivalent of our P2 model. We used the default Ra-LPS available from CHARMM-GUI^97^ and built an asymmetric membrane with 81 LPS on the outer leaflet and 246 POPE in the inner leaflet. Three systems were modeled for energy calculation (zero, one, and five polymyxins).

Each of the three systems was first equilibrated, before proceeding to a steered MD procedure and energy calculations by means of umbrella sampling and WHAM.^105^ Equilibration was led following CHARMM-GUI^97^ protocol adapted to the Martini force field^111^ (similar to the one for the CHARMM force field^45^ described above) and terminated by a 2 μs-long equilibration run. The steered MD simulation consisted of a 10 ns relaxation with no bias applied, followed by an additional 10 ns at close to the equilibrium value (15 ions in the cylinder). The run to reach a minimal value along the CV was 1 μs. Then, the umbrella sampling procedure was applied for all of the systems between 0 and 20 ions in the cylinder. Simulations times are reported in Table 5.

**Table 5:** Simulation Times for Production Runs of the Different Systems^a^

number of polymyxins	equilibration	steered MD	umbrella sampling	number of windows
0	2 μs	>1 μs	3 μs/window	73
1	1 μs	>1 μs	2 μs/window	72
5	1 μs	>1 μs	3 μs/window	81

a Note that the equilibration time with polymyxin molecules of 1 μs comes after inserting the drugs on the equilibrated membrane.

### Packing Defect

We applied Packmem^112^ in four distinct scenarios: (1) equilibrated membranes without any external stress, (2) membranes under global stress, (3) membranes subjected to minimal local stress, and (4) membrane exposed to extreme local stress. In scenario 1, we evaluated the packing defect coefficients over 100 ns, with frames separated by 20 ps, resulting in over 300,000 packing defects in each membrane. For scenario 2, the coefficients were assessed over 1200 ns, with frames spaced 200 ps apart, and a total of more than 210,000 packing defects. In scenarios 3 and 4, we analyzed the packing defect during the last 50 ns of umbrella sampling for the corresponding windows, with frames separated by 20 ps and over 150,000 packing defects detected. For packing defect size estimation, we used the third carbon branching the fifth and sixth lipid tails to distinguish between deep and shallow packing defects, focusing on deep packing defects buried within the fatty acid layer, particularly those associated with a central tail in our LPS model.

### Membrane Curvature and Fluctuations

We applied the SuAVE package^46^ in the same four scenarios considered for the computation of the packing defect. SuAVE was used to analyze the shape and fluctuations of equilibrated and stressed membranes by computing the distribution of surface angles. It generates a map based on user-selected atoms from both membrane leaflets, which captures membrane undulations. Here, we selected the phosphorus atom in POPE and the carbons involved in the β (1–6) linkage of the lipid A headgroup. We avoided selecting the phosphorus atoms in the LPS molecules, as it is advisable to use atoms in more stable positions, which might not be the case of the lipid A phosphorus atoms that are side groups of the lipid A disaccharide. SuAVE calculates the normal to this map across a discretized grid and determines the angle between this normal and the *z*-axis.

### Density Profiles

We used GROMACS^108^ density to compute the density profiles of our systems. Beforehand, we checked that the mean and Gaussian curvature of every system could be considered null and that the amplitude of the membrane undulatory fluctuations do not exceed those of similar systems. We used the GROMACS trjconv tool to translate the membrane so that the origin along the *z*-axis corresponds to the center of geometry of the last aliphatic carbon of POPE and LPS molecules. Particularly, we computed the density profiles of the membranes under global stress.

We analyzed the membranes under global stress and used the density profiles as a criterion for checking system equilibrium, to analyze the difference in the depth at which water penetrates the membranes under local and global stress and to compute the proportion of LPS flipping under global stress. For the analyses of the membranes under global stress, we used an equivalent of 100 ns of simulations over four replicates, for which we observed signs of equilibration. For the analyses of the membranes under local stress, we used the last 100 ns of the given umbrella window.

### Proportion of LPS Flipping

To compute the proportion of LPS flipping, the density profile was integrated. The density is centered in 0, which corresponds to the center of geometry of the methyl groups of LPS and POPE. The proportion of flipped LPS is the integral of all values lower than 0 divided by the integral of the whole profile. Only the last block of the trajectory was used (25 ns) for each replicate. Errors are computed as the standard error of the four replicas.

### Area per Lipid

To compute the area per lipid, we applied the FatSlim package.^48^ The same reference atoms and segment of trajectory were used for the area per lipid as for packing defect analysis.

### Lipid Displacement

To compute the number of phosphorus atoms inside the cylinder as a function of the local surface density of divalent ions, we relied on the PLUMED library^109^ to implement the analysis. We computed the number of phosphorus atoms with the DENSITY keyword in the very same fashion as our CV is implemented. We did that for each window of the umbrella sampling procedures using the last 100 ns of the trajectory.

### Insertion Depth

The analysis of the insertion depth was computed using the MDAnalysis library.^109^ We computed, for each umbrella sampling window, the distance between the center of geometry of polymyxin to that of lipid A phosphate groups, using the last 100 ns of the trajectory.

### Density Maps

We computed the density maps showing the freezing of multiple chemical groups using GROMACS densmap.^108^

### Root Mean Square Fluctuations

The analysis of the RMSF of the phosphate groups was computed using the MDAnalysis library,^113,114^ with which we analyzed every umbrella sampling window.

## Data Availability

Initial configurations, input parameters, and topologies are available at https://zenodo.org/doi/10.5281/zenodo.13486347.

## Supporting Information Available

Analysis of the density profiles of relevant groups for the global stress; distribution of the surface angle of the membranes in the absence of stress and under global stress; depth at which water molecules penetrate the LPS leaflet; distribution of the area per lipid of LPS molecules; data used to compute the percentage of LPS flipping; defect size constant for the local stress; distribution of the minimal distance between phosphorus atoms of LPS molecules; orientation of LPS molecule when a local stress is applied; RMSF of lipid A phosphorus atoms when a local stress is applied; measurement of the reaction of the system to the moving biasing potential during the steered MD simulation; and histograms associated with the umbrella sampling procedure (PDF)
Steered MD simulation for P2 illustrating the collective variable (MP4)
Steered MD simulation for P2 showing the collective motion of phosphorus atoms with calcium ions, and the absence of such co-motion for the aliphatic carbons (MP4)

## Abbreviations

MDR: multidrug-resistant
IM: inner membrane
OM: outer membrane
PE: phosphatidylethanolamine
PG: phosphatidylglycerol
CL: cardiolipins
LPS: lipopolysaccharides
DAB: l-diaminobutyric acid
PMB: polymyxin B
PME: polymyxin E, colistin
MD: molecular dynamics
PEtN: phosphoethanolamine
Ara-4N: 4-aminoarabinose
GalN: galactosamine
EDTA: ethylenediaminetetraacetic
LOESS: local regression smoothing
RMSF: root mean squared fluctuations
WHAM: weighted histogram analysis method
SPME: smooth particle mesh Ewald.

## References

[ref1] WHO publishes list of bacteria for which new antibiotics are urgently needed. https://www.who.int/news/item/27-02-2017-who-publishes-list-of-bacteria-for-which-new-antibiotics-are-urgently-needed (accessed 2024–03–28).

[ref2] Hawkey P. M. Multidrug-Resistant Gram-Negative Bacteria: A Product of Globalization. Journal of Hospital Infection 2015, 89 (4), 241–247. 10.1016/j.jhin.2015.01.008. 25737092

[ref3] Bassetti M.; Garau J. Current and Future Perspectives in the Treatment of Multidrug-Resistant Gram-Negative Infections. J. Antimicrob. Chemother. 2021, 76 (Supplement_4), iv23–iv37. 10.1093/jac/dkab352. 34849997 PMC8632738

[ref4] Renwick M. J.; Brogan D. M.; Mossialos E. A Systematic Review and Critical Assessment of Incentive Strategies for Discovery and Development of Novel Antibiotics. J. Antibiot (Tokyo) 2016, 69 (2), 73–88. 10.1038/ja.2015.98. 26464014 PMC4775540

[ref5] Kostyanev T.; Bonten M. J. M.; O’Brien S.; Steel H.; Ross S.; François B.; Tacconelli E.; Winterhalter M.; Stavenger R. A.; Karlén A.; Harbarth S.; Hackett J.; Jafri H. S.; Vuong C.; MacGowan A.; Witschi A.; Angyalosi G.; Elborn J. S.; deWinter R.; Goossens H. The Innovative Medicines Initiative’s New Drugs for Bad Bugs Programme: European Public–Private Partnerships for the Development of New Strategies to Tackle Antibiotic Resistance. J. Antimicrob. Chemother. 2016, 71 (2), 290–295. 10.1093/jac/dkv339. 26568581

[ref6] Ribeiro da Cunha B.; Fonseca L. P.; Calado C. R. C. Antibiotic Discovery: Where Have We Come from, Where Do We Go?. Antibiotics (Basel) 2019, 8 (2), 45 10.3390/antibiotics8020045. 31022923 PMC6627412

[ref7] Wu E. L.; Engström O.; Jo S.; Stuhlsatz D.; Yeom M. S.; Klauda J. B.; Widmalm G.; Im W. Molecular Dynamics and NMR Spectroscopy Studies of E. Coli Lipopolysaccharide Structure and Dynamics. Biophys. J. 2013, 105 (6), 1444–1455. 10.1016/j.bpj.2013.08.002. 24047996 PMC3785875

[ref8] Lundstedt E.; Kahne D.; Ruiz N. Assembly and Maintenance of Lipids at the Bacterial Outer Membrane. Chem. Rev. 2021, 121 (9), 5098–5123. 10.1021/acs.chemrev.0c00587. 32955879 PMC7981291

[ref9] Sun J.; Rutherford S. T.; Silhavy T. J.; Huang K. C. Physical Properties of the Bacterial Outer Membrane. Nat. Rev. Microbiol 2022, 20 (4), 236–248. 10.1038/s41579-021-00638-0. 34732874 PMC8934262

[ref10] Nikaido H. Molecular Basis of Bacterial Outer Membrane Permeability Revisited. Microbiol. Mol. Biol. Rev. 2003, 67 (4), 593–656. 10.1128/MMBR.67.4.593-656.2003. 14665678 PMC309051

[ref11] Vergalli J.; Bodrenko I. V.; Masi M.; Moynié L.; Acosta-Gutiérrez S.; Naismith J. H.; Davin-Regli A.; Ceccarelli M.; van den Berg B.; Winterhalter M.; Pagès J.-M. Porins and Small-Molecule Translocation across the Outer Membrane of Gram-Negative Bacteria. Nat. Rev. Microbiol 2020, 18 (3), 164–176. 10.1038/s41579-019-0294-2. 31792365

[ref12] Needham B. D.; Trent M. S. Fortifying the Barrier: The Impact of Lipid A Remodelling on Bacterial Pathogenesis. Nat. Rev. Microbiol 2013, 11 (7), 467–481. 10.1038/nrmicro3047. 23748343 PMC6913092

[ref13] Lam N. H.; Ma Z.; Ha B.-Y. Electrostatic Modification of the Lipopolysaccharide Layer: Competing Effects of Divalent Cations and Polycationic or Polyanionic Molecules. Soft Matter 2014, 10 (38), 7528–7544. 10.1039/C4SM01262C. 25109281

[ref14] Kim S.; Patel D. S.; Park S.; Slusky J.; Klauda J. B.; Widmalm G.; Im W. Bilayer Properties of Lipid A from Various Gram-Negative Bacteria. Biophys. J. 2016, 111 (8), 1750–1760. 10.1016/j.bpj.2016.09.001. 27760361 PMC5071556

[ref15] Velkov T.; Roberts K. D.; Nation R. L.; Thompson P. E.; Li J. Pharmacology of Polymyxins: New Insights into an “old” Class of Antibiotics. Future Microbiol. 2013, 8 (6), 711–724. 10.2217/fmb.13.39. 23701329 PMC3852176

[ref16] Whitfield C.; Williams D. M.; Kelly S. D. Lipopolysaccharide O-Antigens—Bacterial Glycans Made to Measure. J. Biol. Chem. 2020, 295 (31), 10593–10609. 10.1074/jbc.REV120.009402. 32424042 PMC7397119

[ref17] Jefferies D.; Shearer J.; Khalid S. Role of O-Antigen in Response to Mechanical Stress of the E. Coli Outer Membrane: Insights from Coarse-Grained MD Simulations. J. Phys. Chem. B 2019, 123 (17), 3567–3575. 10.1021/acs.jpcb.8b12168. 30971088

[ref18] Evans M. E.; Feola D. J.; Rapp R. P. Polymyxin B Sulfate and Colistin: Old Antibiotics for Emerging Multiresistant Gram-Negative Bacteria. Ann. Pharmacother. 1999, 33 (9), 960–967. 10.1345/aph.18426. 10492501

[ref19] Sakura N.; Itoh T.; Uchida Y.; Ohki K.; Okimura K.; Chiba K.; Sato Y.; Sawanishi H. The Contribution of the *N*-Terminal Structure of Polymyxin B Peptides to Antimicrobial and Lipopolysaccharide Binding Activity. Bull. Chem. Soc. Jpn. 2004, 77 (10), 1915–1924. 10.1246/bcsj.77.1915.

[ref20] Kanazawa K.; Sato Y.; Ohki K.; Okimura K.; Uchida Y.; Shindo M.; Sakura N. Contribution of Each Amino Acid Residue in Polymyxin B_3_ to Antimicrobial and Lipopolysaccharide Binding Activity. Chem. Pharm. Bull. 2009, 57 (3), 240–244. 10.1248/cpb.57.240. 19252313

[ref21] Velkov T.; Thompson P. E.; Nation R. L.; Li J. Structure–Activity Relationships of Polymyxin Antibiotics. J. Med. Chem. 2010, 53 (5), 1898–1916. 10.1021/jm900999h. 19874036 PMC2907661

[ref22] Kimura Y.; Matsunaga H.; Vaara M. Polymyxin B-Octapeptide and Polymyxin B-Heptapeptide Are Potent Outer-Membrane Permeability-Increasing Agents. J. Antibiot. 1992, 45 (5), 742–749. 10.7164/antibiotics.45.742. 1624376

[ref23] Katz M.; Tsubery H.; Kolusheva S.; Shames A.; Fridkin M.; Jelinek R. Lipid Binding and Membrane Penetration of Polymyxin B Derivatives Studied in a Biomimetic Vesicle System. Biochem. J. 2003, 375, 405–413. 10.1042/bj20030784. 12848621 PMC1223683

[ref24] World Health Organization; WHO Advisory Group on Integrated Surveillance of Antimicrobial Resistance (AGISAR). Critically Important Antimicrobials for Human Medicine: Ranking of Antimicrobial Agents for Risk Management of Antimicrobial Resistance Due to Non-Human Use, 5th rev.; World Health Organization: Geneva, 2017.

[ref25] Warren H.; Kania S.; Siber G. Binding the Neutralization of Bacterial Lipopolysaccharide by Colistin Nonapeptide. Antimicrob. Agents Chemother. 1985, 28 (1), 107–112. 10.1128/AAC.28.1.107. 2412488 PMC176319

[ref26] Vaara M. The Outer-Membrane Permeability-Increasing Action of Linear Analogs of Polymyxin-B Nonapeptide. Drug Exp. Clin. Res. 1991, 17 (9), 437–444. 1822436

[ref27] Berglund N. A.; Piggot T. J.; Jefferies D.; Sessions R. B.; Bond P. J.; Khalid S. Interaction of the Antimicrobial Peptide Polymyxin B1 with Both Membranes of E. Coli: A Molecular Dynamics Study. PLoS Comput. Biol. 2015, 11 (4), e1004180 10.1371/journal.pcbi.1004180. 25885324 PMC4401565

[ref28] Trimble M. J.; Mlynarcik P.; Kolar M.; Hancock R. E. W. Polymyxin: Alternative Mechanisms of Action and Resistance. Cold Spring Harb. Perspect. Med. 2016, 6 (10), a025288 10.1101/cshperspect.a025288. 27503996 PMC5046685

[ref29] Dupuy F. G.; Pagano I.; Andenoro K.; Peralta M. F.; Elhady Y.; Heinrich F.; Tristram-Nagle S. Selective Interaction of Colistin with Lipid Model Membranes. Biophys. J. 2018, 114 (4), 919–928. 10.1016/j.bpj.2017.12.027. 29490251 PMC5985005

[ref30] Cetuk H.; Anishkin A.; Scott A. J.; Rempe S. B.; Ernst R. K.; Sukharev S. Partitioning of Seven Different Classes of Antibiotics into LPS Monolayers Supports Three Different Permeation Mechanisms through the Outer Bacterial Membrane. Langmuir 2021, 37 (4), 1372–1385. 10.1021/acs.langmuir.0c02652. 33449700

[ref31] Sabnis A.; Hagart K. L. H.; Klockner A.; Becce M.; Evans L. E.; Furniss R. C. D.; Mavridou D. A.; Murphy R.; Stevens M. M.; Davies J. C.; Larrouy-Maumus G. J.; Clarke T. B.; Edwards A. M. Colistin Kills Bacteria by Targeting Lipopolysaccharide in the Cytoplasmic Membrane. eLife 2021, 10, e65836 10.7554/eLife.65836. 33821795 PMC8096433

[ref32] Khondkerg A.; Dhaliwal A. K.; Saem S.; Mahmood A.; Fradin C.; Moran-Mirabal J.; Rheinstadte M. C. Membrane Charge and Lipid Packing Determine Polymyxin-Induced Membrane Damage. Commun. Biol. 2019, 2, 67 10.1038/s42003-019-0297-6. 30793045 PMC6379423

[ref33] Wallace S. J.; Li J.; Nation R. L.; Prankerd R. J.; Velkov T.; Boyd B. J. Self-Assembly Behavior of Colistin and Its Prodrug Colistin Methanesulfonate: Implications for Solution Stability and Solubilization. J. Phys. Chem. B 2010, 114 (14), 4836–4840. 10.1021/jp100458x. 20302384 PMC2881163

[ref34] Hancock I. C.; Meadow P. M. The Extractable Lipids of Pseudomonas Aeruginosa. Biochimica et Biophysica Acta (BBA) - Lipids and Lipid Metabolism 1969, 187 (3), 366–379. 10.1016/0005-2760(69)90010-1. 4981667

[ref35] Adams M. D.; Nickel G. C.; Bajaksouzian S.; Lavender H.; Murthy A. R.; Jacobs M. R.; Bonomo R. A. Resistance to Colistin in *Acinetobacter Baumannii* Associated with Mutations in the PmrAB Two-Component System. Antimicrob. Agents Chemother. 2009, 53 (9), 3628–3634. 10.1128/AAC.00284-09. 19528270 PMC2737849

[ref36] Han M.-L.; Velkov T.; Zhu Y.; Roberts K. D.; Le Brun A. P.; Chow S. H.; Gutu A. D.; Moskowitz S. M.; Shen H.-H.; Li J. Polymyxin-Induced Lipid A Deacylation in Pseudomonas Aeruginosa Perturbs Polymyxin Penetration and Confers High-Level Resistance. ACS Chem. Biol. 2018, 13 (1), 121–130. 10.1021/acschembio.7b00836. 29182311

[ref37] Ma W.; Jiang X.; Dou Y.; Zhang Z.; Li J.; Yuan B.; Yang K. Biophysical Impact of Lipid A Modification Caused by Mobile Colistin Resistance Gene on Bacterial Outer Membranes. J. Phys. Chem. Lett. 2021, 12 (48), 11629–11635. 10.1021/acs.jpclett.1c03295. 34817187

[ref38] Pelletier M. R.; Casella L. G.; Jones J. W.; Adams M. D.; Zurawski D. V.; Hazlett K. R. O.; Doi Y.; Ernst R. K. Unique Structural Modifications Are Present in the Lipopolysaccharide from Colistin-Resistant Strains of *Acinetobacter Baumannii*. Antimicrob. Agents Chemother. 2013, 57 (10), 4831–4840. 10.1128/AAC.00865-13. 23877686 PMC3811424

[ref39] Soon R. L.; Nation R. L.; Harper M.; Adler B.; Boyce J. D.; Tan C.-H.; Li J.; Larson I. Effect of Colistin Exposure and Growth Phase on the Surface Properties of Live *Acinetobacter Baumannii* Cells Examined by Atomic Force Microscopy. Int. J. Antimicrob. Agents 2011, 38 (6), 493–501. 10.1016/j.ijantimicag.2011.07.014. 21925844 PMC3433558

[ref40] Santos D. E. S.; Pol-Fachin L.; Lins R. D.; Soares T. A. Polymyxin Binding to the Bacterial Outer Membrane Reveals Cation Displacement and Increasing Membrane Curvature in Susceptible but Not in Resistant Lipopolysaccharide Chemotypes. J. Chem. Inf. Model. 2017, 57 (9), 2181–2193. 10.1021/acs.jcim.7b00271. 28805387

[ref41] Fu L.; Wan M.; Zhang S.; Gao L.; Fang W. Polymyxin B Loosens Lipopolysaccharide Bilayer but Stiffens Phospholipid Bilayer. Biophys. J. 2020, 118 (1), 138–150. 10.1016/j.bpj.2019.11.008. 31812355 PMC6950770

[ref42] Li J.; Beuerman R.; Verma C. S. Mechanism of Polyamine Induced Colistin Resistance through Electrostatic Networks on Bacterial Outer Membranes. Biochim. Biophys. Acta-Biomembr. 2020, 1862 (9), 183297 10.1016/j.bbamem.2020.183297. 32339485

[ref43] Jiang X.; Sun Y.; Yang K.; Yuan B.; Velkov T.; Wang L.; Li J. Coarse-Grained Simulations Uncover Gram-Negative Bacterial Defense against Polymyxins by the Outer Membrane. Comp. Struct. Biotechnol. J. 2021, 19, 3885–3891. 10.1016/j.csbj.2021.06.051. PMC8441625 34584634

[ref44] Nguyen H. L.; Linh H. Q.; Krupa P.; La Penna G.; Li M. S. Amyloid β Dodecamer Disrupts the Neuronal Membrane More Strongly than the Mature Fibril: Understanding the Role of Oligomers in Neurotoxicity. J. Phys. Chem. B 2022, 126 (20), 3659–3672. 10.1021/acs.jpcb.2c01769. 35580354 PMC9150093

[ref45] Yoo J.; Aksimentiev A. New Tricks for Old Dogs: Improving the Accuracy of Biomolecular Force Fields by Pair-Specific Corrections to Non-Bonded Interactions. Phys. Chem. Chem. Phys. 2018, 20 (13), 8432–8449. 10.1039/C7CP08185E. 29547221 PMC5874203

[ref46] Santos D. E. S.; Pontes F. J. S.; Lins R. D.; Coutinho K.; Soares T. A. SuAVE: A Tool for Analyzing Curvature-Dependent Properties in Chemical Interfaces. J. Chem. Inf. Model. 2020, 60 (2), 473–484. 10.1021/acs.jcim.9b00569. 31508962

[ref47] Rice A.; Rooney M. T.; Greenwood A. I.; Cotten M. L.; Wereszczynski J. Lipopolysaccharide Simulations Are Sensitive to Phosphate Charge and Ion Parameterization. J. Chem. Theory Comput. 2020, 16 (3), 1806–1815. 10.1021/acs.jctc.9b00868. 32023054 PMC7257439

[ref48] Buchoux S. FATSLiM: A Fast and Robust Software to Analyze MD Simulations of Membranes. Bioinformatics 2017, 33 (1), 133–134. 10.1093/bioinformatics/btw563. 27578804

[ref49] Ma H.; Irudayanathan F. J.; Jiang W.; Nangia S. Simulating Gram-Negative Bacterial Outer Membrane: A Coarse Grain Model. J. Phys. Chem. B 2015, 119 (46), 14668–14682. 10.1021/acs.jpcb.5b07122. 26374325

[ref50] Jefferies D.; Hsu P.-C.; Khalid S. Through the Lipopolysaccharide Glass: A Potent Antimicrobial Peptide Induces Phase Changes in Membranes. Biochemistry 2017, 56 (11), 1672–1679. 10.1021/acs.biochem.6b01063. 28248490

[ref51] Marvin H. J.; Ter Beest M. B.; Witholt B. Release of Outer Membrane Fragments from Wild-Type Escherichia Coli and from Several E. Coli Lipopolysaccharide Mutants by EDTA and Heat Shock Treatments. J. Bacteriol. 1989, 171 (10), 5262–5267. 10.1128/jb.171.10.5262-5267.1989. 2507517 PMC210360

[ref52] Vaara M. Agents That Increase the Permeability of the Outer Membrane. Microbiol Rev. 1992, 56 (3), 395–411. 10.1128/mr.56.3.395-411.1992. 1406489 PMC372877

[ref53] Prachayasittikul V.; Isarankura-Na-Ayudhya C.; Tantimongcolwat T.; Nantasenamat C.; Galla H.-J. EDTA-Induced Membrane Fluidization and Destabilization: Biophysical Studies on Artificial Lipid Membranes. ABBS 2007, 39 (11), 901–913. 10.1111/j.1745-7270.2007.00350.x. 17989882

[ref54] Clifton L. A.; Skoda M. W. A.; Le Brun A. P.; Ciesielski F.; Kuzmenko I.; Holt S. A.; Lakey J. H. Effect of Divalent Cation Removal on the Structure of Gram-Negative Bacterial Outer Membrane Models. Langmuir 2015, 31 (1), 404–412. 10.1021/la504407v. 25489959 PMC4295546

[ref55] Clifton L. A.; Holt S. A.; Hughes A. V.; Daulton E. L.; Arunmanee W.; Heinrich F.; Khalid S.; Jefferies D.; Charlton T. R.; Webster J. R. P.; Kinane C. J.; Lakey J. H. An Accurate In Vitro Model of the E. Coli Envelope. Angew. Chem., Int. Ed. 2015, 54 (41), 11952–11955. 10.1002/anie.201504287. PMC4600229 26331292

[ref56] Vazdar K.; Tempra C.; Olżyńska A.; Biriukov D.; Cwiklik L.; Vazdar M. Stealthy Player in Lipid Experiments? EDTA Binding to Phosphatidylcholine Membranes Probed by Simulations and Monolayer Experiments. J. Phys. Chem. B 2023, 127 (24), 5462–5469. 10.1021/acs.jpcb.3c03207. 37307026 PMC10291544

[ref57] Fu L.; Li X.; Zhang S.; Dong Y.; Fang W.; Gao L. Polymyxins Induce Lipid Scrambling and Disrupt the Homeostasis of Gram-Negative Bacteria Membrane. Biophys. J. 2022, 121 (18), 3486–3498. 10.1016/j.bpj.2022.08.007. 35964158 PMC9515121

[ref58] Piggot T. J.; Holdbrook D. A.; Khalid S. Electroporation of the E. Coli and S. Aureus Membranes: Molecular Dynamics Simulations of Complex Bacterial Membranes. J. Phys. Chem. B 2011, 115 (45), 13381–13388. 10.1021/jp207013v. 21970408

[ref59] Piggot T. J.; Holdbrook D. A.; Khalid S. Conformational Dynamics and Membrane Interactions of the E. Coli Outer Membrane Protein FecA: A Molecular Dynamics Simulation Study. Biochimica et Biophysica Acta (BBA) - Biomembranes 2013, 1828 (2), 284–293. 10.1016/j.bbamem.2012.08.021. 22960041

[ref60] Lopez C. A.; Zgurskaya H.; Gnanakaran S. Molecular Characterization of the Outer Membrane of Pseudomonas Aeruginosa. Biochim. Biophys. Acta-Biomembr. 2020, 1862 (3), 183151 10.1016/j.bbamem.2019.183151. 31846648

[ref61] Guo L.; Lim K. B.; Poduje C. M.; Daniel M.; Gunn J. S.; Hackett M.; Miller S. I. Lipid A Acylation and Bacterial Resistance against Vertebrate Antimicrobial Peptides. Cell 1998, 95 (2), 189–198. 10.1016/S0092-8674(00)81750-X. 9790526

[ref62] Bader M. W.; Navarre W. W.; Shiau W.; Nikaido H.; Frye J. G.; McClelland M.; Fang F. C.; Miller S. I. Regulation of *Salmonella Typhimurium* Virulence Gene Expression by Cationic Antimicrobial Peptides. Mol. Microbiol. 2003, 50 (1), 219–230. 10.1046/j.1365-2958.2003.03675.x. 14507376

[ref63] Olaitan A. O.; Morand S.; Rolain J.-M. Mechanisms of Polymyxin Resistance: Acquired and Intrinsic Resistance in Bacteria. Front. Microbiol. 2014, 5, 643 10.3389/fmicb.2014.00643. 25505462 PMC4244539

[ref64] Lay W. K.; Miller M. S.; Elcock A. H. Optimizing Solute–Solute Interactions in the GLYCAM06 and CHARMM36 Carbohydrate Force Fields Using Osmotic Pressure Measurements. J. Chem. Theory Comput. 2016, 12 (4), 1401–1407. 10.1021/acs.jctc.5b01136. 26967542 PMC5082696

[ref65] Schneider T.; Muellera A.; Miess H.; Gross H. Cyclic Lipopeptides as Antibacterial Agents - Potent Antibiotic Activity Mediated by Intriguing Mode of Actions. Int. J. Med. Microbiol. 2014, 304 (1), 37–43. 10.1016/j.ijmm.2013.08.009. 24119568

[ref66] Mohapatra S. S.; Dwibedy S. K.; Padhy I. Polymyxins, the Last-Resort Antibiotics: Mode of Action, Resistance Emergence, and Potential Solutions. J. Biosci. 2021, 46 (3), 85 10.1007/s12038-021-00209-8. 34475315 PMC8387214

[ref67] Manioglu S.; Modaresi S. M.; Ritzmann N.; Thoma J.; Overall S. A.; Harms A.; Upert G.; Luther A.; Barnes A. B.; Obrecht D.; Müller D. J.; Hiller S. Antibiotic Polymyxin Arranges Lipopolysaccharide into Crystalline Structures to Solidify the Bacterial Membrane. Nat. Commun. 2022, 13 (1), 6195 10.1038/s41467-022-33838-0. 36271003 PMC9587031

[ref68] Prakaash D.; González A. B.; Waterhouse F.; Khalid S. Characterization of Antimicrobial Peptide Interactions with a Realistically Phase Separated Model of the E. Coli Outer Membrane via Large-Scale Simulations. Biophys. J. 2024, 123 (3), 328a 10.1016/j.bpj.2023.11.2004.

[ref69] Bai G.; Yi H.-B.; Li H.-J.; Xu J.-J. Hydration Characteristics of Ca^2+^ and Mg^2+^: A Density Functional Theory, Polarized Continuum Model and Molecular Dynamics Investigation. Mol. Phys. 2013, 111 (4), 553–568. 10.1080/00268976.2012.737035.

[ref70] Xu H.-T.; Zhang N.; Li M.-R.; Zhang F.-S. Comparison of the Ionic Effects of Ca 2 + and Mg 2 + on Nucleic Acids in Liquids. J. Mol. Liq. 2021, 344, 117781 10.1016/j.molliq.2021.117781.

[ref71] Sharma P.; Ayappa K. G. A Molecular Dynamics Study of Antimicrobial Peptide Interactions with the Lipopolysaccharides of the Outer Bacterial Membrane. J. Membr. Biol. 2022, 255 (6), 665–675. 10.1007/s00232-022-00258-6. 35960325

[ref72] Jiang X.; Han M.; Tran K.; Patil N. A.; Ma W.; Roberts K. D.; Xiao M.; Sommer B.; Schreiber F.; Wang L.; Velkov T.; Li J. An Intelligent Strategy with All-Atom Molecular Dynamics Simulations for the Design of Lipopeptides against Multidrug- Resistant Pseudomonas Aeruginosa. J. Med. Chem. 2022, 65 (14), 10001–10013. 10.1021/acs.jmedchem.2c00657. 35786900

[ref73] Gonzalez-Fernandez C. A.; Bringas E.; Oostenbrink C.; Ortiz I. In Silico Investigation and Surmounting of Lipopolysaccharide Barrier in Gram-Negative Bacteria: How Far Has Molecular Dynamics Come?. Comp. Struct. Biotechnol. J. 2022, 20, 5886–5901. 10.1016/j.csbj.2022.10.039. PMC9636410 36382192

[ref74] Hancock R. E. W.; Chapple D. S. Peptide Antibiotics. Antimicrob. Agents Chemother. 1999, 43 (6), 1317–1323. 10.1128/AAC.43.6.1317. 10348745 PMC89271

[ref75] Deris Z. Z.; Swarbrick J. D.; Roberts K. D.; Azad M. A. K.; Akter J.; Horne A. S.; Nation R. L.; Rogers K. L.; Thompson P. E.; Velkov T.; Li J. Probing the Penetration of Antimicrobial Polymyxin Lipopeptides into Gram-Negative Bacteria. Bioconjugate Chem. 2014, 25 (4), 750–760. 10.1021/bc500094d. PMC3993906 24635310

[ref76] LaPorte D. C.; Rosenthal K. S.; Storm D. R. Inhibition of Escherichia Coli Growth and Respiration by Polymyxin B Covalently Attached to Agarose Beads. Biochemistry 1977, 16 (8), 1642–1648. 10.1021/bi00627a019. 192271

[ref77] Zdrazil B.; Felix E.; Hunter F.; Manners E. J.; Blackshaw J.; Corbett S.; de Veij M.; Ioannidis H.; Lopez D. M.; Mosquera J. F.; Magarinos M. P.; Bosc N.; Arcila R.; Kizilören T.; Gaulton A.; Bento A. P.; Adasme M. F.; Monecke P.; Landrum G. A.; Leach A. R. The ChEMBL Database in 2023: A Drug Discovery Platform Spanning Multiple Bioactivity Data Types and Time Periods. Nucleic Acids Res. 2024, 52 (D1), D1180–D1192. 10.1093/nar/gkad1004. 37933841 PMC10767899

[ref78] O’Boyle N. M.; Banck M.; James C. A.; Morley C.; Vandermeersch T.; Hutchison G. R. Open Babel: An Open Chemical Toolbox. J. Cheminform 2011, 3 (1), 33 10.1186/1758-2946-3-33. 21982300 PMC3198950

[ref79] Jo S.; Kim T.; Iyer V. G.; Im W. CHARMM-GUI: A Web-Based Graphical User Interface for CHARMM. J. Comput. Chem. 2008, 29 (11), 1859–1865. 10.1002/jcc.20945. 18351591

[ref80] Kim S.; Lee J.; Jo S.; Brooks C. L.; Lee H. S.; Im W. CHARMM-GUI Ligand Reader and Modeler for CHARMM Force Field Generation of Small Molecules: CHARMM-GUI Ligand Reader and Modeler for CHARMM Force Field Generation of Small Molecules. J. Comput. Chem. 2017, 38 (21), 1879–1886. 10.1002/jcc.24829. 28497616 PMC5488718

[ref81] Pristovšek P.; Kidrič J. Solution Structure of Polymyxins B and E and Effect of Binding to Lipopolysaccharide: An NMR and Molecular Modeling Study. J. Med. Chem. 1999, 42 (22), 4604–4613. 10.1021/jm991031b. 10579822

[ref82] Graham J. A.; Essex J. W.; Khalid S. PyCGTOOL: Automated Generation of Coarse-Grained Molecular Dynamics Models from Atomistic Trajectories. J. Chem. Inf. Model. 2017, 57 (4), 650–656. 10.1021/acs.jcim.7b00096. 28345910

[ref83] Rice A.; Wereszczynski J. Atomistic Scale Effects of Lipopolysaccharide Modifications on Bacterial Outer Membrane Defenses. Biophys. J. 2018, 114 (6), 1389–1399. 10.1016/j.bpj.2018.02.006. 29590596 PMC5883967

[ref84] Kučerka N.; Papp-Szabo E.; Nieh M.-P.; Harroun T. A.; Schooling S. R.; Pencer J.; Nicholson E. A.; Beveridge T. J.; Katsaras J. Effect of Cations on the Structure of Bilayers Formed by Lipopolysaccharides Isolated from Pseudomonas Aeruginosa PAO1. J. Phys. Chem. B 2008, 112 (27), 8057–8062. 10.1021/jp8027963. 18549267

[ref85] Edrington T. C.; Kintz E.; Goldberg J. B.; Tamm L. K. Structural Basis for the Interaction of Lipopolysaccharide with Outer Membrane Protein H (OprH) from *Pseudomonas Aeruginosa**. J. Biol. Chem. 2011, 286 (45), 39211–39223. 10.1074/jbc.M111.280933. 21865172 PMC3234746

[ref86] Lerouge I.; Vanderleyden J. O-Antigen Structural Variation: Mechanisms and Possible Roles in Animal/Plant–Microbe Interactions. FEMS Microbiol Rev. 2002, 26 (1), 17–47. 10.1111/j.1574-6976.2002.tb00597.x. 12007641

[ref87] Ongwae G. M.; Morrison K. R.; Allen R. A.; Kim S.; Im W.; Wuest W. M.; Pires M. M. Broadening Activity of Polymyxin by Quaternary Ammonium Grafting. ACS Infect. Dis. 2020, 6 (6), 1427–1435. 10.1021/acsinfecdis.0c00037. 32212668 PMC7293573

[ref88] Li A.; Schertzer J. W.; Yong X. Molecular Dynamics Modeling of Pseudomonas Aeruginosa Outer Membranes. Phys. Chem. Chem. Phys. 2018, 20 (36), 23635–23648. 10.1039/C8CP04278K. 30191217 PMC6151269

[ref89] Jiang X.; Yang K.; Han M.-L.; Yuan B.; Li J.; Gong B.; Velkov T.; Schreiber F.; Wang L.; Li J. Outer Membranes of Polymyxin-Resistant *Acinetobacter Baumannii* with Phosphoethanolamine-Modified Lipid A and Lipopolysaccharide Loss Display Different Atomic-Scale Interactions with Polymyxins. ACS Infect. Dis. 2020, 6 (10), 2698–2708. 10.1021/acsinfecdis.0c00330. 32871077 PMC7554230

[ref90] Li J.; Beuerman R.; Verma C. S. Dissecting the Molecular Mechanism of Colistin Resistance in Mcr-1 Bacteria. J. Chem. Inf. Model. 2020, 60 (10), 4975–4984. 10.1021/acs.jcim.0c01051. 33017152

[ref91] Patil N. A.; Ma W.; Jiang X.; He X.; Yu H. H.; Wickremasinghe H.; Wang J.; Thompson P. E.; Velkov T.; Roberts K. D.; Li J. Critical Role of Position 10 Residue in the Polymyxin Antimicrobial Activity. J. Med. Chem. 2023, 66 (4), 2865–2876. 10.1021/acs.jmedchem.2c01915. 36745479

[ref92] Awang T.; Pongprayoon P. The Penetration of Human Defensin 5 (HD5) through Bacterial Outer Membrane: Simulation Studies. J. Mol. Model. 2021, 27 (10), 291 10.1007/s00894-021-04915-w. 34546425

[ref93] Sun Y.; Deng Z.; Jiang X.; Yuan B.; Yang K. Interactions between Polymyxin B and Various Bacterial Membrane Mimics: A Molecular Dynamics Study. Colloid Surf. B-Biointerfaces 2022, 211, 112288 10.1016/j.colsurfb.2021.112288. 34942463

[ref94] Clifton L. A.; Skoda M. W. A.; Daulton E. L.; Hughes A. V.; Le Brun A. P.; Lakey J. H.; Holt S. A. Asymmetric Phospholipid: Lipopolysaccharide Bilayers; a Gram-Negative Bacterial Outer Membrane Mimic. J. R. Soc. Interface. 2013, 10 (89), 20130810 10.1098/rsif.2013.0810. 24132206 PMC3808558

[ref95] Bello G.; Bodin A.; Lawrence M. J.; Barlow D.; Mason A. J.; Barker R. D.; Harvey R. D. The Influence of Rough Lipopolysaccharide Structure on Molecular Interactions with Mammalian Antimicrobial Peptides. Biochimica et Biophysica Acta (BBA) - Biomembranes 2016, 1858 (2), 197–209. 10.1016/j.bbamem.2015.11.007. 26592318

[ref96] Vieira T.; Dos Santos C. A.; De Jesus Bertani A. M.; Costa G. L.; Campos K. R.; Sacchi C. T.; Cunha M. P. V.; Carvalho E.; Da Costa A. J.; De Paiva J. B.; Rubio M. D. S.; Camargo C. H.; Tiba-Casas M. R. Polymyxin Resistance in Salmonella: Exploring Mutations and Genetic Determinants of Non-Human Isolates. Antibiotics 2024, 13 (2), 110 10.3390/antibiotics13020110. 38391496 PMC10885896

[ref97] Lee J.; Patel D. S.; Ståhle J.; Park S.-J.; Kern N. R.; Kim S.; Lee J.; Cheng X.; Valvano M. A.; Holst O.; Knirel Y. A.; Qi Y.; Jo S.; Klauda J. B.; Widmalm G.; Im W. CHARMM-GUI *Membrane Builder* for Complex Biological Membrane Simulations with Glycolipids and Lipoglycans. J. Chem. Theory Comput. 2019, 15 (1), 775–786. 10.1021/acs.jctc.8b01066. 30525595

[ref98] Chen H. D.; Groisman E. A. The Biology of the PmrA/PmrB Two-Component System: The Major Regulator of Lipopolysaccharide Modifications. Annu. Rev. Microbiol. 2013, 67, 83–112. 10.1146/annurev-micro-092412-155751. 23799815 PMC8381567

[ref99] Dalebroux Z. D.; Miller S. I. Salmonellae PhoPQ Regulation of the Outer Membrane to Resist Innate Immunity. Curr. Opin Microbiol 2014, 17, 106–113. 10.1016/j.mib.2013.12.005. 24531506 PMC4043142

[ref100] Huang J.; Li C.; Song J.; Velkov T.; Wang L.; Zhu Y.; Li J. Regulating Polymyxin Resistance in Gram-Negative Bacteria: Roles of Two-Component Systems PhoPQ and PmrAB. Future Microbiol. 2020, 15 (6), 445–459. 10.2217/fmb-2019-0322. 32250173 PMC7236789

[ref101] Zhou Z.; Ribeiro A. A.; Lin S.; Cotter R. J.; Miller S. I.; Raetz C. R. H. Lipid A Modifications in Polymyxin-Resistant Salmonella Typhimurium. J. Biol. Chem. 2001, 276 (46), 43111–43121. 10.1074/jbc.M106960200. 11535603

[ref102] Gibbons H. S.; Kalb S. R.; Cotter R. J.; Raetz C. R. H. Role of Mg^2+^ and pH in the Modification of *Salmonella* Lipid A after Endocytosis by Macrophage Tumour Cells. Mol. Microbiol. 2005, 55 (2), 425–440. 10.1111/j.1365-2958.2004.04409.x. 15659161

[ref103] Raetz C. R. H.; Reynolds C. M.; Trent M. S.; Bishop R. E. Lipid A Modification Systems in Gram-Negative Bacteria. Annu. Rev. Biochem. 2007, 76 (1), 295–329. 10.1146/annurev.biochem.76.010307.145803. 17362200 PMC2569861

[ref104] Guo L.; Lim K. B.; Gunn J. S.; Bainbridge B.; Darveau R. P.; Hackett M.; Miller S. I. Regulation of Lipid A Modifications by *Salmonella Typhimurium* Virulence Genes *phoP-phoQ*. Science 1997, 276 (5310), 250–253. 10.1126/science.276.5310.250. 9092473

[ref105] Bauer D. Danijoo/WHAM: Bugfixes 2021, 10.5281/ZENODO.1488597.

[ref106] Bonomi M.; Bussi G.; Camilloni C.; Tribello G. A.; Banáš P.; Barducci A.; Bernetti M.; Bolhuis P. G.; Bottaro S.; Branduardi D.; Capelli R.; Carloni P.; Ceriotti M.; Cesari A.; Chen H.; Chen W.; Colizzi F.; De S.; De La Pierre M.; Donadio D.; Drobot V.; Ensing B.; Ferguson A. L.; Filizola M.; Fraser J. S.; Fu H.; Gasparotto P.; Gervasio F. L.; Giberti F.; Gil-Ley A.; Giorgino T.; Heller G. T.; Hocky G. M.; Iannuzzi M.; Invernizzi M.; Jelfs K. E.; Jussupow A.; Kirilin E.; Laio A.; Limongelli V.; Lindorff-Larsen K.; Löhr T.; Marinelli F.; Martin-Samos L.; Masetti M.; Meyer R.; Michaelides A.; Molteni C.; Morishita T.; Nava M.; Paissoni C.; Papaleo E.; Parrinello M.; Pfaendtner J.; Piaggi P.; Piccini G.; Pietropaolo A.; Pietrucci F.; Pipolo S.; Provasi D.; Quigley D.; Raiteri P.; Raniolo S.; Rydzewski J.; Salvalaglio M.; Sosso G. C.; Spiwok V.; Šponer J.; Swenson D. W. H.; Tiwary P.; Valsson O.; Vendruscolo M.; Voth G. A.; White A. The PLUMED consortium. Promoting Transparency and Reproducibility in Enhanced Molecular Simulations. Nat. Methods 2019, 16 (8), 670–673. 10.1038/s41592-019-0506-8. 31363226

[ref107] Essmann U.; Perera L.; Berkowitz M. L.; Darden T.; Lee H.; Pedersen L. G. A Smooth Particle Mesh Ewald Method. J. Chem. Phys. 1995, 103 (19), 8577–8593. 10.1063/1.470117.

[ref108] Abraham M. J.; Murtola T.; Schulz R.; Páll S.; Smith J. C.; Hess B.; Lindahl E. GROMACS: High Performance Molecular Simulations through Multi-Level Parallelism from Laptops to Supercomputers. SoftwareX 2015, 1–2, 19–25. 10.1016/j.softx.2015.06.001.

[ref109] Tribello G. A.; Bonomi M.; Branduardi D.; Camilloni C.; Bussi G. PLUMED 2: New Feathers for an Old Bird. Comput. Phys. Commun. 2014, 185 (2), 604–613. 10.1016/j.cpc.2013.09.018.

[ref110] Hess B.; Bekker H.; Berendsen H. J. C.; Fraaije J. G. E. M. LINCS: A Linear Constraint Solver for Molecular Simulations. J. Comput. Chem. 1997, 18 (12), 1463–1472. 10.1002/(SICI)1096-987X(199709)18:12<1463::AID-JCC4>3.0.CO;2-H.

[ref111] De Jong D. H.; Singh G.; Bennett W. F. D.; Arnarez C.; Wassenaar T. A.; Schäfer L. V.; Periole X.; Tieleman D. P.; Marrink S. J. Improved Parameters for the Martini Coarse-Grained Protein Force Field. J. Chem. Theory Comput. 2013, 9 (1), 687–697. 10.1021/ct300646g. 26589065

[ref112] Gautier R.; Bacle A.; Tiberti M. L.; Fuchs P. F.; Vanni S.; Antonny B. PackMem: A Versatile Tool to Compute and Visualize Interfacial Packing Defects in Lipid Bilayers. Biophys. J. 2018, 115 (3), 436–444. 10.1016/j.bpj.2018.06.025. 30055754 PMC6084522

[ref113] Michaud-Agrawal N.; Denning E. J.; Woolf T. B.; Beckstein O. MDAnalysis: A Toolkit for the Analysis of Molecular Dynamics Simulations. J. Comput. Chem. 2011, 32 (10), 2319–2327. 10.1002/jcc.21787. 21500218 PMC3144279

[ref114] Gowers R.; Linke M.; Barnoud J.; Reddy T.; Melo M.; Seyler S.; Domański J.; Dotson D.; Buchoux S.; Kenney I.; Beckstein O. MDAnalysis: A Python Package for the Rapid Analysis of Molecular Dynamics Simulations; Austin, TX, 2016; pp 98–105. 10.25080/Majora-629e541a-00e.

